# Spatial ecology of the *Capnocytophaga* genus in the human oral cavity

**DOI:** 10.1128/spectrum.03626-25

**Published:** 2026-04-30

**Authors:** Jonathan J. Giacomini, Julian Torres-Morales, Floyd E. Dewhirst, Gary G. Borisy, Jessica L. Mark Welch

**Affiliations:** 1 ADA Forsyth Institute, Somerville, Massachusetts, USA; 2 Harvard School of Dental Medicine 124048 , Boston, Massachusetts, USA; 3 Marine Biological Laboratory 42700, Woods Hole, Massachusetts, USA; The Ohio State University College of Dentistry, Columbus, Ohio, USA

**Keywords:** oral microbiome, metapangenomics, habitat specialization, microbial ecology, *Capnocytophaga*

## Abstract

The human oral microbiome, a complex ecosystem of niche-specific communities influenced by local ecological factors, plays a critical role in health and disease. *Capnocytophaga* species are prevalent in the human mouth, often abundant in dental plaque and linked to both commensalism and pathogenicity, motivating a detailed study of their ecological and functional diversity. This study employs metapangenomics to reveal *Capnocytophaga* strain-level distributions and functional adaptations across distinct sites in the human oral cavity. Pangenomic, phylogenetic, and average nucleotide identity analyses enabled classification of unnamed genomes and identified 13 groups, of which 8 include validly named species, and the remainder are named using Human Microbial Taxon (HMT) designations in the Human Oral Microbiome Database (HOMD; https://www.homd.org/). Mapping metagenomic reads to the pangenome revealed a strong preference of most *Capnocytophaga* genomes for dental plaque (both supra- and subgingival), yet identified strain-level variants of *C. sputigena*, *C. gingivalis*, *C. granulosa*, and *C. leadbetteri* detected more often on the tongue. Among dental plaque-abundant taxa, functional analyses uncovered two clades: one with cbb3-type cytochrome oxidase that is tied to enhanced denitrification and could help the organism adapt to hypoxic zones, and another with bd-type ubiquinol oxidase, more suited to aerobic metabolism. Carbohydrate and amino acid metabolism pathways also differed between these clades. These findings identify metabolic adaptations that may underlie sub-specialization within the plaque habitat and highlight the strain-level diversity of *Capnocytophaga,* including low-prevalence strains that are preferentially detected in sites outside the primary plaque habitat of this taxon.

**IMPORTANCE:**

Understanding the ecological roles of *Capnocytophaga* in the oral microbiome is critical for deciphering its contributions to health and disease, including periodontal and systemic infections. This metapangenomics study reveals a pronounced specialization by *Capnocytophaga* to dental plaque (including supragingival, subgingival, and periodontal pockets) and identifies metabolic adaptations, such as distinct respiratory, carbohydrate, and amino acid pathways, that may drive niche-specific survival. These findings support the site-specialist hypothesis and enhance our understanding of oral microbial community structure, laying a foundation for future research into microbial interactions and targeted therapies for oral health.

## INTRODUCTION

The site-specialist hypothesis posits that microbial taxa adapt to specialized niches, forming unique communities shaped by ecological conditions (1). In the human mouth, there are distinct sites, such as supragingival plaque, tongue dorsum, and buccal mucosa, forming a heterogeneous landscape harboring diverse microbial communities (2–7). For example, both dental plaque and the biofilm on the tongue dorsum are multilayered biofilms, featuring steep oxygen gradients—particularly pronounced in subgingival plaque and periodontal pockets—creating microhabitats that influence microbial distributions and interactions, impacting oral health and disease (1, 8–10). Analyzing how bacteria specialize in these environments is crucial for understanding their ecological roles and contributions to microbiome structure in the human mouth.

*Capnocytophaga* species are Gram-negative capnophilic bacteria, often abundant in dental plaque (1, 6). These bacteria are known to leverage CO_2_-rich conditions (11–13), ferment carbohydrates to acetate and degrade proteins for amino acids, enabling efficient energy and nutrient acquisition in the nutrient-rich biofilm of plaque (14) and the anaerobic environments of subgingival plaque and periodontal pockets (50). Common species, including *C. gingivalis*, *C. sputigena*, and *C. ochracea*, are linked to periodontal disease (15, 16) and endodontic infections (17), as well as systemic infections (18), such as sepsis (19), lung co-infections (20), and associations with cancer (21, 22), yet often coexist commensally in healthy hosts (23). This dual nature complicates the understanding of their ecological and functional roles in health and disease. The genus also includes many unnamed species, reflecting its broad diversity and the ongoing challenge of fully classifying its members (23, 24). Clarifying the taxonomy of *Capnocytophaga* is essential to better understand its niche preferences, metabolic adaptations, and contributions to oral health and disease.

Metapangenomics, by integrating pangenomic and metagenomic analyses, enables high-resolution mapping of microbial distributions, as well as taxonomic refinement and analysis of functional diversity in complex ecosystems (7, 25–29). Less precise methods, such as 16S rRNA sequencing, often fail to distinguish closely related species (30), including the closely related *C. gingivalis* and *C. granulosa* (6), obscuring their distributions across oral niches and across individuals and limiting ecological insights. Metapangenomics has recently been applied to several groups of oral bacteria, resolving strain-level differences in niche-specific adaptations (7, 25–28). A similar approach can be used to reveal *Capnocytophaga* distributions and functional traits at the strain level.

This study employs metapangenomics to investigate the distribution and functional traits of *Capnocytophaga* species and their strains across various sites in the human mouth. The goals were to (i) clarify the genomic diversity within this group of bacteria, (ii) determine their habitat preferences, and (iii) explore functional traits that may shape their roles in the human oral microbiome. These findings highlight the breadth of *Capnocytophaga* diversity in the human mouth and offer new insights into their functional diversity.

## MATERIALS AND METHODS

Analyses were conducted using a metapangenomics pipeline based primarily on Anvi’o version 8 (31) and Python v3.10 (32) as described below. Figures were generated using R version 4.4.2 (33).

### Reference genome collection

Compiling a set of high-quality reference genomes is a critical first step in metapangenomics as it establishes a baseline for comparing genetic diversity and distribution across microbial populations. To create a reference genome collection representing the diversity of *Capnocytophaga* in the human oral microbiome, we retrieved the GenBank accession IDs (GCAs) for all publicly available RefSeq genomes from genus *Capnocytophaga* from the National Center for Biotechnology Information (NCBI) database (downloaded on 14-11-2024). To supplement underrepresented *Capnocytophaga* species in RefSeq, we added GCAs for reference genomes from the Genome Taxonomy Database (GTDB R220, downloaded 14-01-2025 from https://gtdb.ecogenomic.org) that were not present in RefSeq. We then filtered the set of RefSeq and GTDB genomes to remove genomes that were isolated from non-human hosts, resulting in a total of 150 genomes, covering 9 named species in NCBI and including genomes classified only to the genus level. To download the selected genomes, we used the NCBI data sets program to retrieve metadata and ftp links based on GCAs. Details about the genomes, including their genus, species, strain, BioSample, BioProject, isolation host, isolation site, RefSeq status, type strain, disease association, and submitter ID, can be found in Supplemental data: Table S1.

We further refined the set of reference genomes, requiring each genome to have ≥90% completeness and <5% contamination (or redundancy) as analyzed by CheckM2 (34) (see Supplemental data: Table S2), and no more than 98% average nucleotide identity (ANI) with any other genome using a greedy algorithm (see Supplemental data: Table S3), prioritizing the choice of complete genomes or type strains. This dereplication threshold was selected to minimize division of metagenomic reads between nearly identical genomes, which can artificially split read recruitment and skew abundance estimates, as demonstrated in prior metapangenomic analyses of oral bacteria (28). To estimate pairwise ANI values, we used the Anvi’o program *anvi-compute-genome-similarity* with the parameters “--program pyANI” and “--method ANIb.” Overall, these quality control and dereplication steps resulted in a set of 117 high-quality dereplicated reference genomes representing the known diversity of *Capnocytophaga* found in the human microbiome.

### Pangenome construction

A pangenome captures the full range of genetic variation within known genomes from a taxonomic group, enabling the study of functional and ecological differences across diverse samples. Using Anvi'o, we constructed a pangenome following previously developed methods (25, 27–29). First, we used *anvi-script-reformat-fasta* to replace non-canonical nucleotide letters with “N” and remove from each reference genome all contigs with a length less than 300 nucleotides. We then converted each genome into an Anvi'o-compatible contigs database using *anvi-gen-contigs-db*. Open reading frames (ORFs), hereafter referred to as genes, were identified by Prodigal v2.6.3. Genes were then annotated with functions using multiple Anvi'o scripts, including *anvi-run-hmms* to find bacterial single-copy genes (Bacteria71 SCG set) (35, 36) with hidden Markov Model (HMM) profiles*, anvi-run-ncbi-cogs* using blastp (v2.10.1+) to annotate with the Cluster of Orthologous Genes (COGs) database (version COG20) (37), and *anvi-run-pfams* and *anvi-run-kegg-kofams* with hmmscan from HMMER (v3.3.1) (38) to functionally annotate with Pfams (v34.0) (39) and KOfams/KEGG Modules (v97.0) (40), respectively. We then used *anvi-pan-genome* to construct the functionally annotated pangenome using blastp to calculate the amino acid-level percent identity between all possible gene pairs. Weak matches were removed using the --minbit criterion of 0.5. The *anvi-pan-genome* program uses a Markov Cluster Algorithm (MCL) to group genes into putatively homologous groups called “gene clusters.” We set the MCL-inflation parameter to 10 as suggested by Anvi’o for comparing very closely related genomes (https://merenlab.org/2016/11/08/pangenomics-v2/). Amino acid sequences within gene clusters were aligned with MUSCLE (v3.8.1551) (41). Finally, we performed hierarchical clustering across gene clusters and genomes using Euclidean distance and Ward linkage. This resulted in a genus-level pangenome showing the distribution of core and accessory genes across 117 *Capnocytophaga* reference genomes.

### Reference genome relatedness: phylogeny and comparison with GTDB

A phylogeny reveals evolutionary relationships among bacterial strains, helping to interpret their genetic diversity and ecological roles. For the pre-dereplicated set of reference genomes (*n* = 150), we constructed a phylogenetic tree based on amino acid sequences of conserved single-copy core genes from the bacterial 71 gene collection (35, 36). We used the Anvi’o program *anvi-get-sequences-for-hmm-hits* to extract amino acid sequences from the pangenome, align protein sequences using MUSCLE (v3.8.1551) (41), and concatenate gene sequences into a fasta file for tree construction. Only genes that occurred in all of the genomes were used for the analysis, which in this case included 12 genes. We trimmed alignments using trimAl (42) with the setting “-gt 0.5” to remove all positions that were gaps in more than 50% of sequences. Maximum likelihood phylogenetic trees were then computed using IQ-TREE (43) with the Whelan and Goldman substitution model (44) and 1,000 bootstrap replicate support. We included a type strain genome for *Flavobacterium johnsoniae* (strain UW101; GCA_000016645.1) to root the trees. An additional phylogenetic analysis, including modifications to gene inclusion criteria and outgroup conditions, is provided in the supplementary text (Supplemental text S2). To compare genomes against the classification in the GTDB, we used GTDB-Tk (version 2.4.1) (45) with classify_wf and the R220 reference data release.

### Distribution of *Capnocytophaga* genomes across human oral sites

We analyzed the distribution of natural populations of *Capnocytophaga* taxa across human oral sites by mapping shotgun metagenomic sequences from the National Institutes of Health Human Microbiome Project (HMP) (46, 47) to our curated set of reference genomes. To obtain data from the HMP portal (https://www.hmpdacc.org/hmp/), we searched for metagenomes using the following parameters: oral sites (buccal mucosa, supragingival plaque, subgingival plaque, dorsum of tongue, hard palate, palatine tonsils, throat, and saliva), Healthy Human Study, fastq files (FASTQ), and whole-genome sequencing (wgs_raw_seq_set). The resulting metagenomes consisted of ~100 bp paired-end reads that were sequenced from samples collected from nine oral sites in phases I and II of the HMP. We performed quality filtering using *iu-filter-quality-minoche* (48), which is based on recommendations from Minoche et al. (49) for Illumina sequencing data. This resulted in a total of 2.5 billion quality-filtered metagenomic short reads from 1,297 samples across nine different oral sites, including three sites in the oral cavity with large sample sizes (the buccal mucosa [*n* = 378], supragingival plaque [*n* = 398], and tongue dorsum [*n* = 428]) and six other sites with smaller sample sizes (subgingival plaque [*n* = 24], keratinized gingiva [*n* = 17], hard palate [*n* = 1], palatine tonsils [*n* = 25], throat [*n* = 18], and saliva [*n* = 8]). Details about the metagenomes, including their BioSample ID, download links, and quality filtering results, can be found in Table S4. Additionally, we downloaded 24 shotgun-sequenced metagenomes sampled from periodontal pockets of subjects diagnosed with periodontal disease (50). These “perio” samples were similarly processed for quality control using *iu-filter-quality-minoche*, results of which can be found in Table S5.

Using Bowtie2 v2.4.1 (51) with the “--very-sensitive,” “--end-to-end,” and “--no-unal” flags, we competitively mapped each quality-filtered metagenomic sample to a fasta file consisting of the 117 selected and dereplicated *Capnocytophaga* reference genomes concatenated together. BAM files were generated from the read alignment data using Samtools v1.9 (52), and the Anvi’o program *anvi-single-profile* was used to generate a read coverage profile for each metagenome. Profiles were then merged for each oral site using *anvi-merge*. We then extracted the mean depth of coverage and breadth of coverage for reads aligned to each genome using *anvi-summarize*.

To accurately assess the presence, abundance, and distribution of bacterial genomes in metagenomic samples from the oral microbiome across habitats, we classified a genome as detected in a metagenomic sample when the whole-genome breadth of coverage (the fraction of the genome covered by at least one read) was at least 50%. This threshold ensures we focus on genomes that are genuinely present and representative in the sample, excluding those that are low-abundance contaminants, spurious matches, or share only a minimal gene set with the microbial community. We then calculated the relative abundance of each detected genome by averaging its depth of coverage across nucleotide positions in which coverage was within the interquartile range (Q2–Q3). Using the Q2–Q3 quartiles of mean depth of coverage provides a more robust measure of mean coverage by mitigating biases from such anomalies, as implemented in the Anvi'o software platform (31) and detailed in its associated documentation (53). To normalize relative abundance, we divided each genome’s mean depth of coverage by the total mean depth of coverage for all detected genomes in a sample, converting this to a percentage. We then summed these relative abundances by genomic groups. To account for potential variability in read depths across metagenomic samples and projects, per-sample read metrics are reported directly in Fig. 2 and 3, enabling visual assessment of their influence on mapping results.

### Functional analysis

Gene functional annotations from Kyoto Encyclopedia of Genes and Genomes (KEGG) and COG enhance our understanding of functional diversity in *Capnocytophaga* by linking specific genes and pathways to metabolic roles, revealing functional differences between taxonomic groups. We predicted the metabolic capabilities of genomes using the Anvi'o script *anvi-estimate-metabolism*. The metabolic pathways considered by this script are those outlined in the KEGG MODULE Database and defined by KEGG Orthologs (KOs) (40, 54). Each KO represents a specific gene function, and a KEGG module is a collection of KOs that work together to complete the steps of a metabolic pathway. To identify COG functional annotations that are differentially enriched or depleted in one set of genomes compared to another, we used the Anvi’o script *anvi-compute-functional-enrichment*. The script associated each gene cluster with the most frequently annotated COG function and generated a frequency table of functions across genomes. An enrichment test was then conducted using a generalized linear model with a logit linkage function to obtain the enrichment score and an adjusted *P* value (q-value).

## RESULTS

### Pangenome, phylogeny, average nucleotide identity, and comparison with GTDB

**Fig 1:**
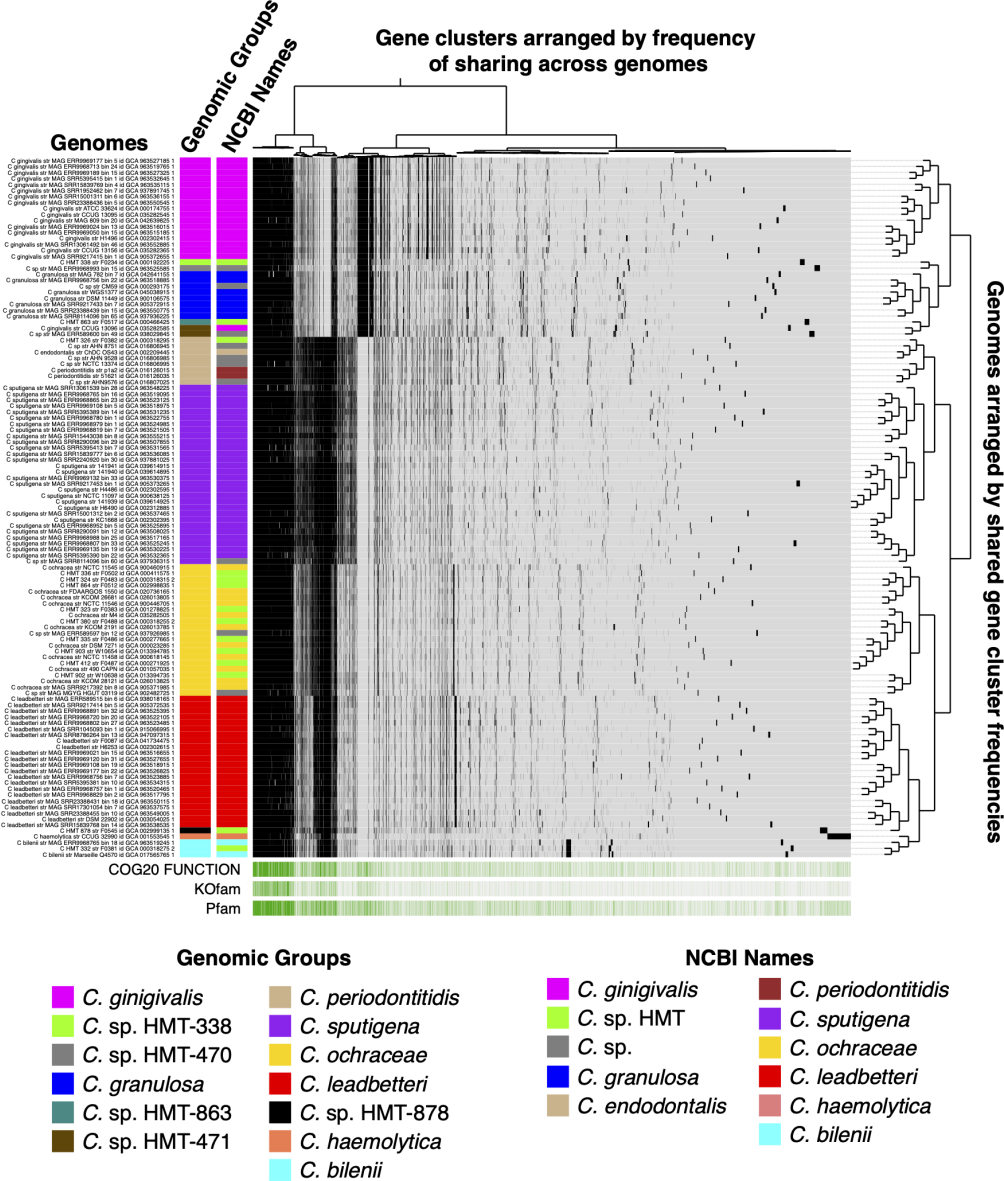
Pangenomic clustering of human-associated *Capnocytophaga* reference genomes. Pangenomic analysis of 117 human-associated *Capnocytophaga* reference genomes from NCBI RefSeq and GTDB databases. ORFs were predicted, and amino acid sequence similarity was calculated using NCBI blastp, followed by clustering of homologous genes with the Markov Cluster Algorithm to form gene clusters. Gene clusters are colored by NCBI species designation and arranged by presence or absence across genomes. Genomes are hierarchically clustered based on gene cluster frequency (number of genomes in which each cluster occurs), with the resulting dendrogram displayed on the right. This analysis reveals distinct species-specific genomic groups, consistent with phylogenomics (Fig. S1 and S2), average nucleotide identity (Fig. S3), and NCBI/GTDB classifications (Table S6), highlighting diversity within the *Capnocytophaga* genus.

Accurate placement of bacterial genomes into biologically meaningful groups is crucial for investigating habitat distribution in the oral cavity. The pangenomic analysis of whole-genome sequences of 117 high-quality, dereplicated *Capnocytophaga* genomes yielded a total of 13,954 gene clusters (Fig. 1). Of these, 341 gene clusters were classified as core (present in all genomes), with the remaining 9,493 comprising accessory genes (present in 2–116 genomes) and 4,120 singletons (present in a single genome). The pangenome showed that genome groups based on shared presence or absence of gene clusters were broadly consistent with phylogenomics (Fig. S1 and S2), pairwise average nucleotide identity (ANI; Fig. S3), and NCBI and GTDB classifications (Table S6). We identified 13 genomic groups, though in some cases, we use “group” loosely, as certain groups include only one or two genomes. Within-group ANI was >95%, except for a single *C. sputigena*-adjacent metagenome-assembled genome (MAG), while between-group ANI was generally <90% (Fig. S3), suggesting that the population genomics of *Capnocytophaga* conforms well to the typically used threshold of 95% ANI. Several unnamed Human Microbial Taxons (HMTs) with divergent 16S rRNA genes were found to be within recognized species groups by this metapangenomic analysis. Thus, of the 13 genomic groups identified in this analysis, 8 genomic groups correspond to validly named species (found in the List of Prokaryotic Names with Standing in Nomenclature, LPSN) and 5 correspond to *Capnocytophaga* sp. with HMT designations in the current version (V4.1) of Human Oral Microbiome Database (HOMD). The genomic group designation assigned for each genome in this study can be found in Table S7. Further clarification of group composition is presented in Supplemental text S2. Collectively, this analysis clarifies genome-relatedness, enabling finer-scale analysis of habitat distributions across the oral cavity.

Based on the pangenomic, phylogenetic, and ANI analyses presented here, together with additional unpublished analyses from our group, we have revised the taxonomy of *Capnocytophaga* in HOMD, merging eight unnamed HMTs into *C. ochracea* and constructing two additional HMTs to incorporate newly defined genomic groups. These revisions are currently implemented in HOMD version V4.1.

### Distribution of *Capnocytophaga* genomes across human oral sites

**Fig 2:**
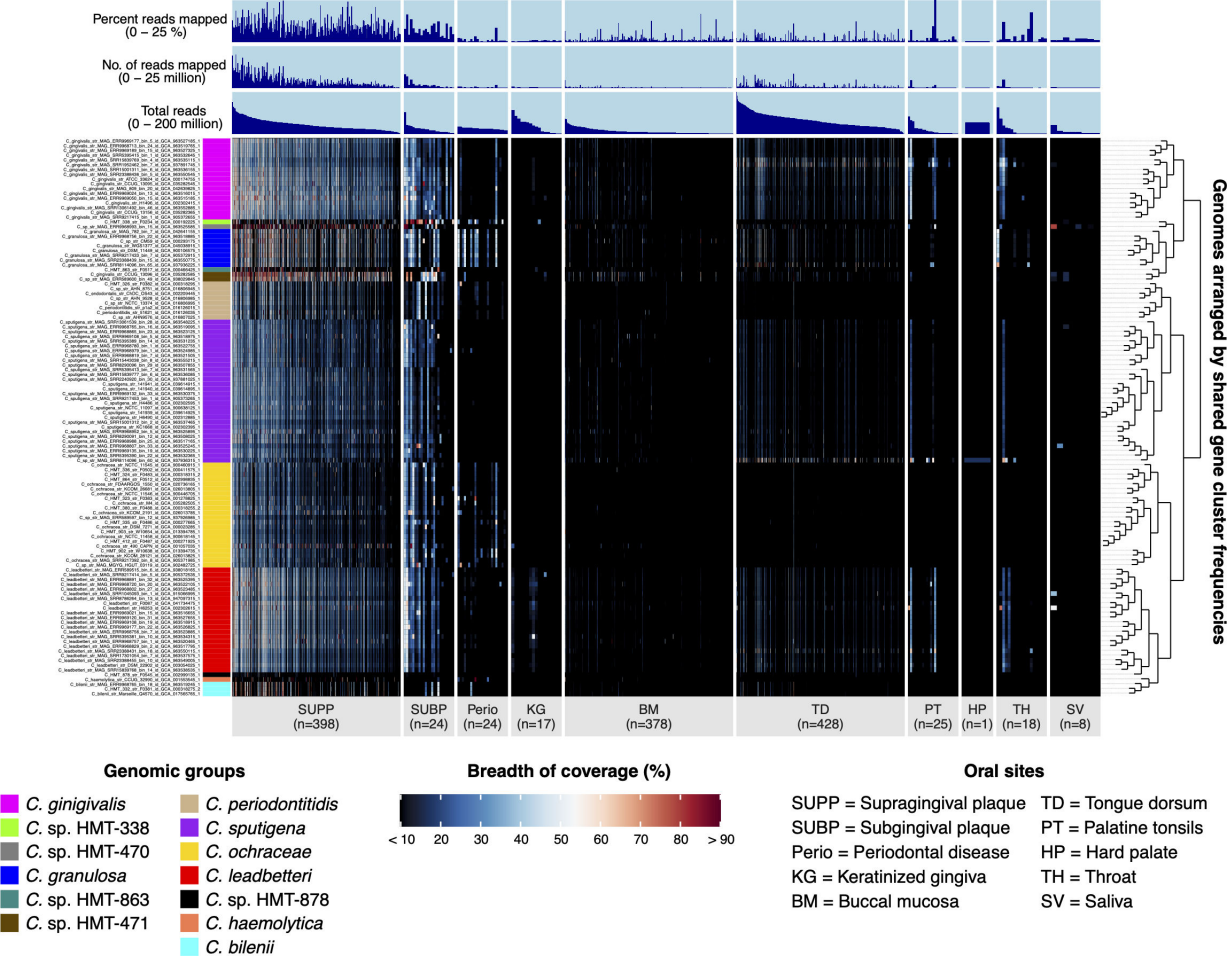
*Capnocytophaga* genome coverage across oral sites. Heatmap of whole-genome breadth of coverage for 117 human-associated *Capnocytophaga* reference genomes resulting from mapping 1,297 HMP metagenomic samples from 9 major oral sites and 24 metagenomic samples from periodontal pockets. This heatmap highlights genome coverage across samples, with broader coverage indicating a higher likelihood of detecting the genome at a specific site. Each row displays the proportion, or breadth, of genome coverage of a genome across all samples. Samples are ordered within each oral site by decreasing number of reads mapped to the set of genomes. From left to right, oral sites are supragingival plaque (SUPP; *n* = 398), subgingival plaque (SUBP; *n* = 24), periodontal pocket (Perio; *n* = 24), keratinized gingiva (KG; *n* = 17), buccal mucosa (BM; *n* = 378), tongue dorsum (TD; *n* = 428 samples), palatine tonsil (PT; *n* = 25), hard palate (HP; *n* = 1), throat (TH; *n* =18), and saliva (SV; *n* = 8). For clearer visualization, we inflated the width of oral sites with small sample sizes. Additional data are shown for “total reads” in each sample, the number of “reads mapped” per sample, and “percent reads mapped” per sample. Genome color bars are included and based on colors designated for genomic groups shown in Fig. 1.

Identifying taxa that occupy distinct niches is essential for unraveling microbial community structure and function. Distribution of taxa across sites within the mouth can be measured using read mapping, which compares metagenomic sequence data to reference genomes and assigns individual reads to matching genome sequences. Mean depth of coverage of a genome indicates how many reads mapped to that genome, whereas breadth of coverage represents the proportion of a genome that recruited reads. Analyzing the breadth of coverage accurately identifies the strain genomes closely related to abundant populations in the sample by distinguishing true overall genome matches from cases where many reads align only to small sections, like mobile genetic elements. Figure 2 shows the breadth of coverage of each reference genome across oral habitats and samples. One striking pattern revealed in Fig. 2 is that 85% of supragingival plaque (SUPP) samples contained at least one *Capnocytophaga* genome exhibiting a breadth of coverage above 50%, indicating a strong genus-level presence in dental plaque. In contrast, only 4% of buccal mucosa (BM) samples and 19% of tongue dorsum (TD) samples showed any *Capnocytophaga* genome with a breadth of coverage above 50%, reflecting a more limited presence in these sites. Like supragingival plaque, many subgingival plaque samples (75%) and periodontal pocket samples (29%) had high breadth of coverage, while keratinized gingiva (12.5%), saliva (4%), palatine tonsils (16%), and throat (17%), like BM and TD, had lower percentages. Habitat distribution patterns remained robust across breadth-of-coverage thresholds of 25%–60% (Table S8; Fig. S9), indicating that meaningful habitat distribution patterns were not sensitively dependent on the breadth of coverage criterion.

These mapping patterns indicate that most *Capnocytophaga* reference genomes closely resemble communities in dental plaque. However, the breadth of coverage heatmap (Fig. 2) highlights outlier MAGs with elevated TD coverage, in contrast to the typical SUPP-dominated profile of their genomic group. These outliers include *C. sputigena* (*n* = 1, GCA_937936315.1), *C. gingivalis* (*n* = 2), *C. granulosa* (*n* = 1), and *C. leadbetteri* (*n* = 1). For example, most *C. sputigena* genomes (*n* = 29) showed rare TD detection (0%–0.4% of samples), but GCA_937936315.1, a MAG classified by GTDB as C. sp937936315, was detected in 10% of TD samples. Outliers for *C. gingivalis*, *C. granulosa*, and *C. leadbetteri* also exhibited this pattern of higher TD detection (Fig. S4).

The high community complexity from which MAGs are constructed significantly increases the likelihood of contamination in the genome assembly, which could result in misleading mapping results. Thus, to determine whether the MAGs that exhibited unusual tongue dorsum prevalence represent genuinely adapted *Capnocytophaga* lineages or chimeric artifacts, we performed detailed contamination checks (see Supplemental text S3 for methods). Specifically, we assessed (i) contig‐level taxonomic classifications, (ii) genome‐wide synteny compared with reference *Capnocytophaga* genomes constructed from isolate cultures, (iii) gene-level coverages from metagenomic read mapping, and (iv) chimeric contamination through taxonomic discordance among gene-level assignments using the Genome UNClutterer (GUNC) (55). For all five TD*-*prevalent MAGs, contigs were consistently classified as *Capnocytophaga*, with most species-level classifications aligning with the expected species group (Fig. S5 and S6). These classifications accounted for over 90% of the sequence length of each MAG (Table S9), indicating that the high (>50%) breadth of coverage findings reported above was not due to contamination with non-*Capnocytophaga* contigs. These five TD-prevalent MAGs also had high levels of pairwise genome alignment with genomes of their respective species groups, ranging from 71% to 94%, on average (Fig. S7). GUNC analysis revealed no evidence of chimerism at the genus level and only very low species-level scores (0.04–0.17), consistent with minor strain-level variation or database effects rather than substantial contamination (Table S10). Lastly, a high proportion (44%–56%) of *Capnocytophaga* genus‐ and species‐specific core genes, as predicted based on the genus-level pangenome (Fig. 1), were detected at a very high breadth of coverage threshold (90%) in tongue dorsum samples (Fig. S8). Overall, we find no evidence of significant contamination that could lead to misleading mapping results, supporting that these MAGs represent authentic strain‐level variants adapted to the tongue dorsum environment.

**Fig 3:**
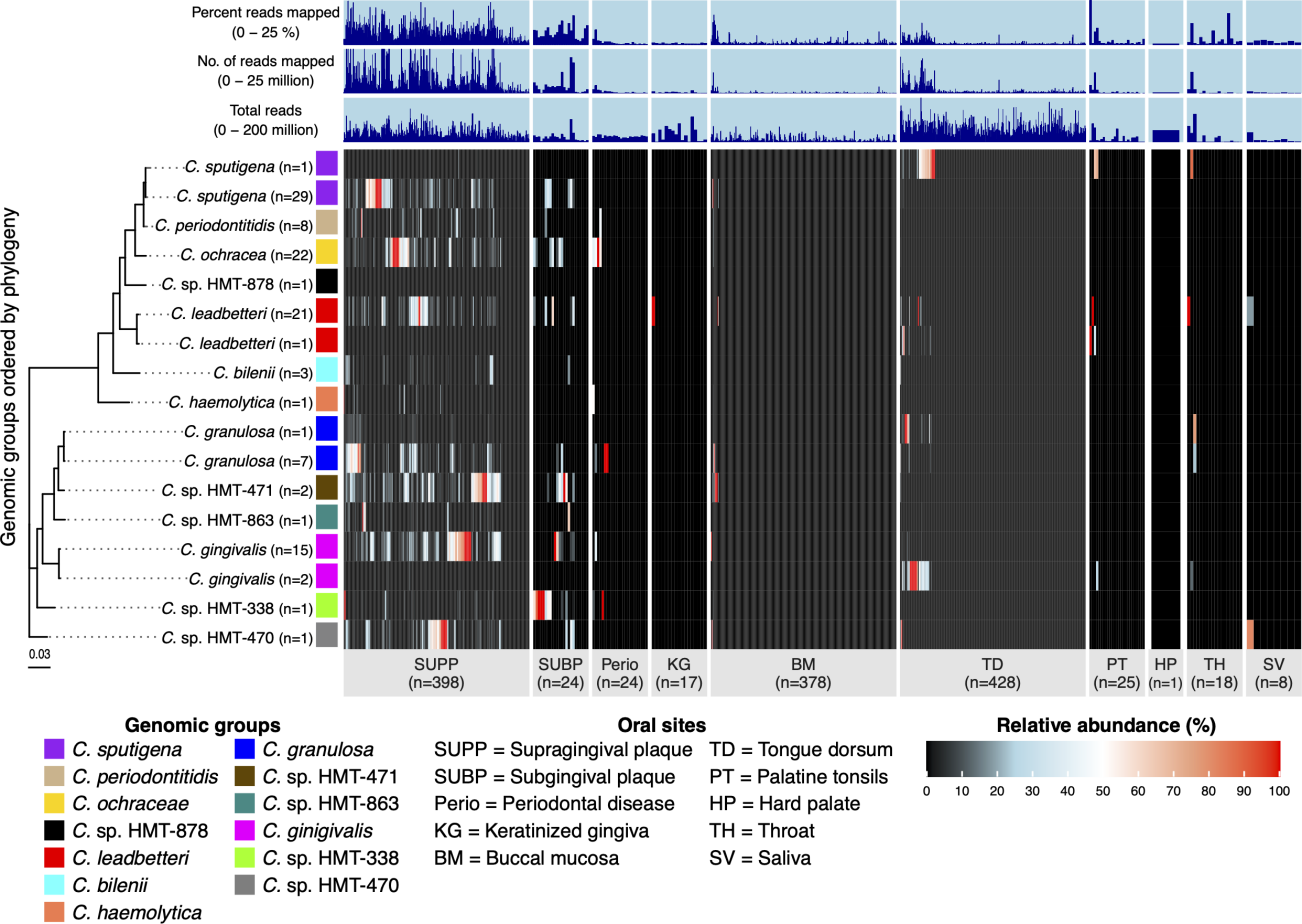
Relative abundance of *Capnocytophaga* genomic groups across oral sites. Heatmap displaying the relative abundance of *Capnocytophaga* genomic groups across 10 oral sites: supragingival plaque (SUPP), subgingival plaque (SUBP), keratinized gingiva (KG), periodontal pocket (Perio), buccal mucosa (BM), tongue dorsum (TD), palatine tonsils (PT), hard palate (HP), throat (TH), and saliva (SV). Rows represent individual genomic groups designated in Fig. 1, while columns correspond to metagenomic samples. The relative abundance of each genomic group is calculated as the sum of relative abundances for all reference genomes within that group, based on the mean depth of coverage across nucleotide positions in the second and third quartiles (Q2–Q3 interquartile range), after ranking nucleotides by depth of coverage. This value is then divided by the total mean coverage of all genomes within a sample. If a genome was undetected (i.e., less than 50% of nucleotides covered at 1×), its coverage was set to zero. Genomic groups (y-axis) are arranged based on a maximum-likelihood phylogenetic tree, constructed using IQ-TREE with amino acid sequences from a 71-gene bacterial collection, with one representative genome selected per group. Samples within oral sites (x-axis) are hierarchically clustered based on Bray-Curtis distances. To improve visualization, the width of oral sites with small sample sizes has been inflated.

To analyze the habitat distribution of both tongue- and plaque-adapted strains, we separated the apparently tongue-adapted outlier strains from plaque-adapted taxa for analysis of their relative abundance. This subdivision resulted in 17 rows in Fig. 3, including 8 groups for validly named species (recognized in LPSN), 5 for HMT designations in HOMD, and the 4 additional outliers showing a tongue dorsum association. Most groups, like *C. sputigena*, *C. granulosa*, *C. ochracea*, *C. bilenii*, *C. periodontitidis*, and *C. leadbetteri*, dominated SUPP and subgingival plaque (SUBP) (60%–80% of *Capnocytophaga* abundance), with rare presence in BM and SV, reflecting a genus-wide plaque bias but low abundance in TD (typically 0%–2%). Outliers of *C. sputigena* (*n* = 1), *C. gingivalis* (*n* = 2), *C. granulosa* (*n* = 1), and *C. leadbetteri* (*n* = 1) showed elevated TD abundance, unlike their group averages, highlighting strain-specific adaptations to unique ecological niches. In periodontitis samples, six groups were abundant in 7 of 24 samples, further reinforcing the genus-wide plaque bias. The relative abundance of the remaining groups varied: *C*. sp. HMT-470, *C*. sp. HMT-471, and *C*. sp. HMT-863 was abundant in SUPP and SUBP; *C*. sp. HMT-338 was abundant in SUBP and in 2 of 24 periodontitis samples; *C. haemolytica* had low relative abundance in SUPP, while *C*. sp. HMT-878 was absent across all sites.

### Functional analysis of *Capnocytophaga* species in the human oral cavity

**Fig 4:**
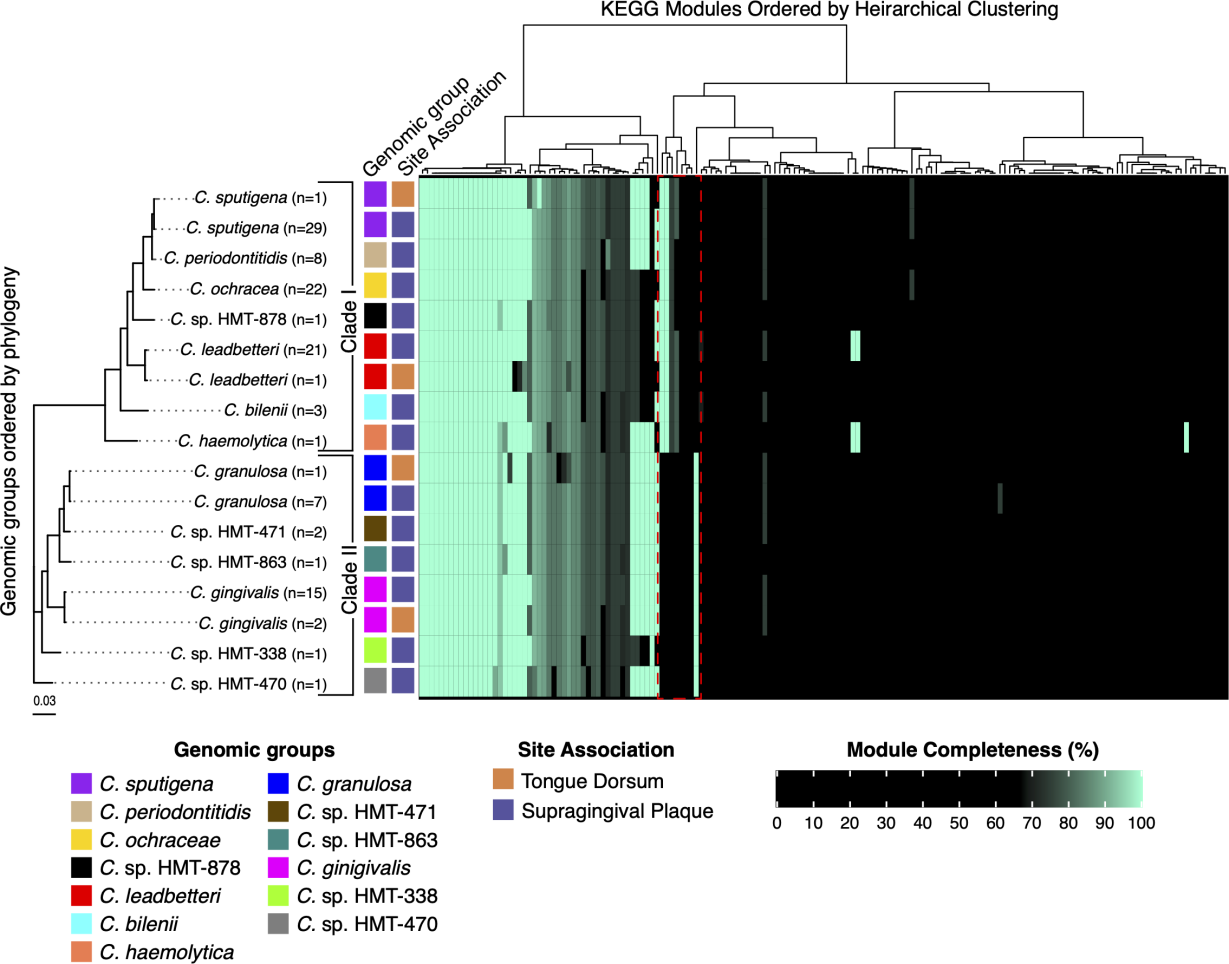
Metabolic diversity of *Capnocytophaga* genomic groups. Heatmap showing the maximum KEGG module completeness (0%–100%) for 17 *Capnocytophaga* genomic groups, calculated using anvi-estimate-metabolism in Anvi’o. Each value reflects the highest module completeness score for any genome within a genomic group. The color scale ranges from black (0%–70%, likely incomplete/non-functional modules) to cyan (>70%–100%, likely complete/functional modules, even if missing some genes). Modules are arranged left to right via hierarchical clustering of completeness scores (Euclidean distances, Ward’s method). Genomic groups (y-axis) are arranged based on a maximum-likelihood phylogenetic tree, forming two distinct clades (designated in the figure as Clade I and Clade II). The phylogenetic tree was constructed using IQ-TREE with amino acid sequences from a 71-gene bacterial collection, with one representative genome selected per group. A dashed red box in the figure highlights key differences in metabolic pathways among the genomic groups. Specifically, the cbb3-type cytochrome c oxidase pathway (M00156) is complete in Clade I genomic groups, while the bd-type ubiquinol oxidase pathway (M00153) is complete in Clade II genomic groups. The galactose Leloir pathway (M00632) is complete in Clade II but only partially present (galE gene only) in Clade I. Additionally, threonine biosynthesis (M00018) is complete in Clade I, whereas cysteine (M00021) and proline (M00970 and M00972) pathways are complete in Clade II. For full module completeness data, refer to Table S9.

Metabolic functional diversity shapes the ability of bacteria to thrive in their ecological niches. To investigate functional diversity among *Capnocytophaga* species, we quantified metabolic pathway completeness across all 117 *Capnocytophaga* genomes and aggregated results to the 17 genomic groups defined in Fig. 3. Details of the results are presented in Table S10 and visualized in a heatmap in Fig. 4, which displays the 17 *Capnocytophaga* genomic groups organized by phylogeny and labeled by species ID and their association with SUPP or TD.

All human oral *Capnocytophaga* genomic groups shared conserved pathways for glucose breakdown to pyruvate (KEGG module: M00001 and M00002), gluconeogenesis (M00003), and ribose synthesis via the non-oxidative pentose phosphate pathway (M00007) and phosphoribosyl pyrophosphate biosynthesis (M00005). Notably, gluconeogenesis (M00003), converting oxaloacetate to fructose-6-phosphate, is complete in all species and includes the *pckA* gene encoding phosphoenolpyruvate carboxykinase, which fixes environmental CO_2_ (e.g., HCO_3_^−^) into intermediary metabolism to produce glucose for energy needs (56). The oxidative pentose phosphate pathway (M00006) for NADPH production, the Entner-Doudoroff pathway (M00008), and glycogen biosynthesis (M00854 and M00855) are consistently incomplete across *Capnocytophaga* species. Lipopolysaccharide (LPS) biosynthesis is largely uniform, with complete pathways for dTDP-L-rhamnose (M00793) and CMP-KDO (M00063), and high completeness (77%–89%) in KDO2-lipid A modules (M00060 and M00866). Fucosamine biosynthesis (M00923), however, is restricted to *C. ochracea* and *C. sputigena* (75% complete), suggesting specialized LPS modifications. Cofactor and vitamin synthesis are mostly complete for coenzyme A (M00120), heme (M00121), biotin (M00123), riboflavin (M00125), folate (M00126), C1-unit interconversion (M00140), and pantothenate (M00119), but incomplete for thiamine (M00127), cobalamin (M00117), molybdopterin (M00925), and niacin/NAD (M00115). Nucleotide synthesis is robust, with complete purine and nearly complete pyrimidine pathways, though pyrimidine degradation is absent except in *C. sputigena* (20% complete). Acetate production (M00579) and a near-complete TCA cycle (M00009, M00010, and M00011) are universal.

We found no differences in KEGG module completeness between the TD-associated genomic groups (i.e., TD-prevalent MAGs) and SUPP-associated groups, suggesting similar metabolic capabilities despite their distinct ecological niches. This result is further supported by a separate COG functional enrichment analysis (Table S11), which indicates there are no COG functions shared exclusively with the TD-associated genomic groups. Additionally, no KEGG modules were present (completeness ≥75%) in the TD-prevalent MAGs but absent (completeness ≤25%) in other genomes within their respective genomic groups. Similarly, functional annotations associated with pangenomic gene clusters present exclusively in TD-prevalent MAGs were not unique but instead were also represented among annotations in plaque-prevalent genomes.

One finding of potential interest for understanding ecological adaptations within this genus is that plaque-preferring *Capnocytophaga* species form two phylogenetically distinct groups with contrasting metabolic adaptations in energy, carbohydrate, and amino acid pathways (Fig. 4). One group, hereafter referred to as Clade I, includes *C. granulosa, C*. sp. HMT-471, *C*. sp. HMT-863, *C. gingivalis, C*. sp. HMT-338, and *C*. sp. HMT-470, which has a complete cbb3-type cytochrome c oxidase pathway (M00156). In contrast, the other group, hereafter referred to as Clade II, includes *C. sputigena, C. periodontitis, C. ochracea, C*. sp. HMT-878*, C. leadbetteri, C. belenii,* and *C. haemolytica* are missing the cbb3-type cytochrome c oxidase pathway and instead have a complete bd-type cytochrome ubiquinol oxidase pathway (M00153). These respiratory differences are supported by a separate COG functional enrichment analysis (Table S11), identifying cbb3 subunits (ccoO, ccoN) in Clade I and bd subunits (appB, appC) in Clade II. Together, these results delineate two *Capnocytophaga* groups in the human oral cavity, distinguished by respiratory mechanisms for oxygen utilization.

Nitrogen metabolism further distinguishes these oxygen-adapted *Capnocytophaga* groups, with Clade I exhibiting enhanced denitrification capabilities. All *Capnocytophaga* species lack nitrate reductase genes, preventing nitrate reduction to nitrite, and most possess nitrite reductases like nrfA and nrfH (nitrite to ammonia) and nirK (nitrite to nitric oxide). However, COG functional enrichment analysis reveals that genomes within Clade I are enriched in azurin, a blue-copper protein aiding denitrification by donating electrons to nitrite reductase, and three nitrous-oxide reductase genes (nosZDL), which are absent in the bd group. Within Clade I, *C. granulosa* also encodes nitric oxide reductase (norB), which is missing from other members of the group.

Carbohydrate and amino acid metabolism pathways also differ between the oxygen-adapted *Capnocytophaga* groups. Clade II possesses a complete Galactose Leloir pathway (M00632), with galK, galM, and galE, enabling full conversion of galactose to UDP-glucose, whereas Clade I has only galE, indicating limited galactose utilization. These groups also show contrasting amino acid acquisition profiles, with Clade I encoding a complete threonine biosynthesis pathway (M00018), which is incomplete in Clade II, while Clade II has complete pathways for cysteine biosynthesis from serine (M00021), proline degradation from glutamate (M00970), and proline synthesis from ornithine (M00972), all consistently incomplete or missing in Clade I. Aromatic amino acid biosynthesis varies across all genomic groups independent of the oxygen adaptations, with tryptophan biosynthesis (M00023) complete in many and entirely lacking in *C. ochracea*, *C. haemolytica*, *C*. sp. HMT-338, and the TD-associated *C. sputigena* and *C. leadbetteri* genomes. Phenylalanine and tyrosine pathways appear incomplete due to the universal absence of aromatic-amino-acid transaminase (tyrB), needed to convert phenylpyruvate to phenylalanine or 4-hydroxy-phenylpyruvate to tyrosine. All genomic groups appear to have a nearly complete Shikimate pathway (M00022), encoding genes required to produce chorismate, the central precursor for the biosynthesis of aromatic amino acids. Only *C. haemolytica* exhibits a complete glycine cleavage system (M00621), a key pathway for glycine catabolism and one-carbon unit production for synthesizing serine, thymidine, and purines (57).

## DISCUSSION

*Capnocytophaga* demonstrated a pronounced ecological specialization in the human oral cavity, favoring dental plaque habitats genus wide. The plaque bias of *Capnocytophaga* likely arises from adaptations to the multilayered biofilm of dental plaque, characterized by steep oxygen gradients (especially in subgingival environments), abundant glycoproteins, and nutrient-rich microhabitats that favor distinct respiratory strategies (8, 58). Functional analysis suggests that the success of human oral *Capnocytophaga* as dental plaque specialists may derive from a conserved metabolic core, including CO_2_ utilization, carbohydrate metabolism, acetate production, cofactor synthesis, and nucleotide pathways, enabling efficient energy use. All detected *Capnocytophaga* species have the genes needed to break down glucose to pyruvate and produce acetate, a fermentation end product. Fermentation, alongside gluconeogenesis and ribose synthesis, ensures a steady supply of energy and building blocks, bypassing incomplete pathways like glycogen storage that may be less critical in plaque. Additionally, all species share complete LPS biosynthesis pathways which reinforce outer membrane integrity, a baseline trait for Gram-negative bacterial survival (59). Amino acid metabolism in *Capnocytophaga* species reflects ecological strategies likely shaped by energy costs (60, 61). All *Capnocytophaga* species in our study can produce chorismate through the shikimate pathway, which is a starting point for building aromatic amino acids like tryptophan, phenylalanine, and tyrosine (62). Many *Capnocytophaga* species have the genes to produce tryptophan, but no human oral *Capnocytophaga* species can produce phenylalanine or tyrosine, as they are missing a key enzyme, suggesting they rely exclusively on external sources for these amino acids via the production of proteolytic enzymes (63, 64). Together, these conserved pathways define a *Capnocytophaga* life strategy likely designed to exploit abundant sugars and CO_2_ in the dental plaque environment while maintaining structural resilience and conserving energy.

Although functional analysis identified a metabolic core common to all *Capnocytophaga* species, it also identified two *Capnocytophaga* sub-groups with different respiratory profiles perhaps suited to different plaque zones. Clade I encodes cbb3-type cytochrome c oxidase, which is known for high oxygen affinity and efficiently reduces O_2_ to H_2_O in low-oxygen settings (65, 66). This process generates a robust proton motive force for ATP synthesis ideal for thriving in deeper, hypoxic layers of dental plaque. Complementary enrichment of nitrite and nitrous oxide reductase genes likely enhances survival in these oxygen-scarce zones by mitigating nitrite accumulation, a trait observed in microaerophilic bacteria like *Pseudomonas* (67, 68). Conversely, Clade II relies on bd-type ubiquinol oxidases, which reduce O_2_ with slightly lower affinity and less efficiency (69), releasing protons directly into the periplasm. This mechanism may support survival near the plaque’s oxygenated surface, where rapid O_2_ shifts occur. Thus, these respiratory distinctions suggest Clade I occupies the anaerobic interior of dental plaque, while Clade II adapts to its more aerobic edges. The two groups further tailor their strategies, with Clade I having a complete pathway to synthesize threonine, while Clade II can produce serine and proline. *Capnocytophaga haemolytica* further stands out with a glycine cleavage system, potentially boosting serine production via one-carbon metabolism (70). Overall, these patterns show how *Capnocytophaga* species conserve energy by skipping expensive pathways when nutrients are plentiful and tailoring amino acid use to potentially enhance survival across different oxygen gradients.

Respiratory and metabolic differences also suggest the likelihood of differential distribution of *Capnocytophaga* within subgingival environments. In our analysis, *Capnocytophaga* genomic groups were prevalent in SUBP and periodontal pockets which are characterized by anaerobic conditions, nutrient limitations, and inflammation (8, 63, 71). The adaptation of Clade I for anaerobic environments (e.g., via cbb3 oxidase and nosZDL genes) may confer specific advantages in subgingival zones by enabling persistence amid nitrite accumulation from dietary or inflammatory sources, efficiently reducing toxic NO₂⁻ to N₂ for detoxification and energy gain (72, 73). Although our data do not directly assess disease states, these functional traits are consistent with prior reports of *Capnocytophaga* presence in periodontal samples (15, 16). Clade II has aerobic adaptations that could favor its survival in the transitional SUPP–SUBP interface. While the absence of distinct distribution patterns between Clade I and Clade II across sampled oral sites suggests that these respiratory adaptations may not confer strong site-level specialization, future studies with increased sample sizes for SUBP and sites with periodontal disease could reveal microhabitat adaptations more readily.

The identification of *Capnocytophaga* genomic groups through metapangenomics clarifies taxonomic relationships, yet the prevalence of groups represented by only one or two genomes reveals substantial unexplored diversity in the human oral microbiome. For instance, the *C*. sp. HMT-471 group, which contains two genomes—an isolate misclassified as *C. gingivalis* in NCBI and a genus-level MAG—highlights how database misclassifications can obscure ecological insights, such as high relative abundance in supragingival and subgingival plaque sites. Similarly, the *C. ochracea* group, which unifies genomes previously labeled as *C*. sp. or HMTs in NCBI, shows that high variability in 16S rRNA led to over-splitting of this species, whereas whole-genome analyses indicate that these genomes form a cohesive cluster based on multiple independent measures of genomic relatedness, including phylogenomics, gene content, and pairwise average nucleotide identity (ANI) >95%. By contrast, in *C. gingivalis*, pangenomics captured subtle genomic divergence that 16S rRNA missed, illustrating how 16S rRNA can either under-split (low resolution) or over-split (high variability) depending on the species.

Strain-level analyses revealed rare exceptions to the plaque tropisms of *Capnocytophaga*. Single genomes from each of *C. sputigena* (GCA_963525245.1), *C. gingivalis* (GCA_937891745.1; GCA_963535115.1), *C. granulosa* (GCA_937936225.1), and *C. leadbetteri* (GCA_963550115.1) showed elevated tongue-dorsum abundance (Fig. 2), diverging from their species’ typical plaque dominance. However, limited sample size (one genome per species) precluded strong functional comparisons between plaque and tongue habitats due to insufficient statistical power. The existence of groups represented by only one or two genomes limits our ability to pinpoint genetic drivers of habitat preferences. These constraints may obscure fine-scale adaptations at the species and strain level, including subtle polymorphisms in adhesion-related genes, regulatory sequences, or accessory elements that could modulate surface interactions and niche specialization without manifesting as broad metabolic divergences detectable through KEGG or COG analyses. This underscores the need for broader genomic sampling to fully elucidate the ecological and functional roles of *Capnocytophaga* in oral health and disease.

### Conclusion

This study refines our understanding of *Capnocytophaga* genomic diversity and confirms the pronounced specialization of this taxon to dental plaque within the oral microbiome, while also revealing intriguing patterns in certain outliers to the tongue dorsum. It reveals metabolic and respiratory adaptations that may underpin their ecological niche. Our findings support the site-specialist hypothesis, enabling targeted investigations into *Capnocytophaga* species interactions with cohabitants and host tissues and providing a foundation for understanding the roles of this genus in oral health and disease progression.

## Data Availability

The raw data used in this study are publicly available at NIH GenBank and RefSeq (https://www.ncbi.nlm.nih.gov/genome/) for genomes and HMP metagenomes from https://www.hmpdacc.org/hmp/; no new data was generated for deposit. Code used for analyses is available on GitHub upon publication of this study (https://github.com/FatherofEverest/Spatial-ecology-of-the-Capnocytophaga-genus-in-the-human-oral-cavity).

## SUPPLEMENTAL MATERIAL

Supplemental figure legends spectrum.03626-25-s0001.docx Legends for Supplemental figures S1 to S9.

Supplemental figures spectrum.03626-25-s0002.pdf Fig. S1 to S9.

Supplemental material spectrum.03626-25-s0003.docx Supplemental text S1 to S3.

Supplemental tables spectrum.03626-25-s0004.xlsx Tables S1 to S14.

ASM does not own the copyrights to Supplemental Material that may be linked to, or accessed through, an article. The authors have granted ASM a non-exclusive, world-wide license to publish the Supplemental Material files. Please contact the corresponding author directly for reuse.
