# Supplementary material for: Spatial ecology of the Capnocytophaga genus in the human oral cavity
Source: Microbiol Spectr. 2026 Apr 30;14(6):e03626-25. doi: 10.1128/spectrum.03626-25 (PMC13228040; doi:10.1128/spectrum.03626-25)
Supplement: Supplemental figure legends — Legends for Supplemental figures S1 to S9. [file spectrum.03626-25-s0001.docx]

**Supplemental Figure Legends**

**Figure S1**: **Phylogenomic tree of *Capnocytophaga* reference genomes.** Maximum-likelihood phylogenomic tree of *Capnocytophaga* reference genomes based on conserved single-copy core genes. Colors denote genomic groups designated in Figure 1, with animal-associated *Capnocytophaga* species distinguished by stripped black boxes to assist in placing unnamed *Capnocytophaga* sp. genomes. The tree was constructed using amino acid sequences from the bacterial 71-gene collection and constructed using IQ-TREE with the Whelan and Goldman (WAG) substitution model and 1000 bootstrap replicate support. The tree is rooted with the type strain genome of *Flavobacterium johnsoniae* (strain UW101; GCA_000016645.1).

**Figure S2: Phylogenomic trees of *Capnocytophaga* reference genomes under varying gene inclusion criteria and outgroup conditions.** Genomes are colored based on their genomic groups designated in Figure 1 or their outgroup status (cat- and dog-associated genomes, or *Flavobacterium* genomes). (A) Maximum likelihood phylogenomic tree based on 12 conserved genes from the bacterial 71 collection, requiring presence in 100% of genomes, with cat- and dog-associated genomes removed. (B–D) Phylogenomic trees based on relaxed gene inclusion thresholds from the bacterial_71 collection, requiring genes to be present in at least 50% (B, 70 genes), 75% (C, 70 genes), or 90% (D, 69 genes) of genomes, with cat- and dog-associated genomes excluded. (E) Unrooted phylogenomic tree constructed from 147 single-copy core genes (SCGs) extracted from the pangenome of 117 human-associated *Capnocytophaga* genomes. All trees were rooted with *Flavobacterium* *johnsoniae* (strain UW101; GCA_000016645.1) as an outgroup.

**Figure S3: Average nucleotide identity (ANI) heatmap of *Capnocytophaga* genomes.** Heatmap of average nucleotide identity (ANI) among *Capnocytophaga* reference genomes, reflecting genome-wide nucleotide similarity. ANI values distinguish genomic groups and corroborate pangenome clustering results. Colors denote genomic groups, with animal-associated *Capnocytophaga* species with black stripes to aid in placing unnamed *Capnocytophaga* sp. genomes. The heatmap color scale represents percent similarity of aligned regions: red indicates 100%, white indicates 95%, and blue indicates 90% or below.

**Figure S4**: **Prevalence ratio of *Capnocytophaga* in supragingival plaque compared to tongue dorsum.** Bar plot of the supragingival plaque (SUPP) to tongue dorsum (TD) prevalence ratio for *Capnocytophaga* reference genomes across selected species. The ratio, plotted on a log10 scale, compares genome prevalence (proportion of samples with ≥50% breadth of coverage) between SUPP and TD sites for *C. sputigena*, *C. gingivalis*, *C. granulosa*, and *C. leadbetteri*. Within each species, genomes are classified (e.g., A, B, C) based on clustering patterns from pangenome analysis, phylogenomic relationships (Figure S1), average nucleotide identity (ANI; Figure S2), and breadth of coverage in metagenomic samples; for example, *C. sputigena* group B includes *Capnocytophaga* sp. MAG_SRR8114096_bin_60. Bars are grouped by species in separate panels, with colors indicating species: purple for *C. sputigena*, magenta for *C. gingivalis*, red for *C. leadbetteri*, and midnight blue for *C. granulosa*. Genomes are ordered by decreasing SUPP:TD ratio within each panel, and a dashed red line at y=1 marks equal prevalence between SUPP and TD.

**Figure S5:** **Contig classification of tongue dorsum *Capnocytophaga* MAGs (NR Database)**. Contig classification analysis of five tongue dorsum-prevalent *Capnocytophaga* metagenome-assembled genomes (MAGs) using the NR protein database in MMSEQS2. **(A)** Genus-level classification: Bar plot showing the proportion of contigs with top hits classified as *Capnocytophaga* (blue) versus other genera (gray) across all five MAGs. **(B)** Species-level classification: Bar plot showing, for contigs classified as *Capnocytophaga* in panel A, the proportion of top hits assigned to specific *Capnocytophaga* species (e.g., *C. sputigena*, *C. gingivalis*), with colors indicating species identity. Classifications are based on top hits from MMSEQS2 taxonomic assignments, supporting the consistency of MAGs with expected *Capnocytophaga* lineages (see Table S8).

**Figure S6:** **Contig classification of tongue dorsum *Capnocytophaga* MAGs (GTDB Database)**. Contig classification analysis of five tongue dorsum-prevalent *Capnocytophaga* metagenome-assembled genomes (MAGs) using the GTDB database in MMSEQS2. **(A)** Genus-level classification: Bar plot showing the proportion of contigs with top hits classified as *Capnocytophaga* (blue) versus other genera (gray) across all five MAGs. **(B)** Species-level classification: Bar plot showing, for contigs classified as *Capnocytophaga* in panel A, the proportion of top hits assigned to specific *Capnocytophaga* species (e.g., *C. sputigena*, *C. gingivalis*), with colors indicating species identity. Classifications are based on top hits from MMSEQS2 taxonomic assignments, corroborating the alignment of MAGs with expected *Capnocytophaga* species groups (see Table S8).

**Figure S7:** **Pairwise alignment coverage heatmap of *Capnocytophaga* genomes**. Heatmap of pairwise alignment coverage among 150 dereplicated *Capnocytophaga* genomes (color bars indicated genomic groups), animal-associated *Capnocytophaga* genomes (black stripes), and the *Flavobacterium johnsoniae* outgroup (strain UW101; GCA_000016645.1; red stripes). The heatmap displays percent alignment coverage (0–100%) between genomes, with colors indicating coverage: red for 100%, white for 50%, and blue for 0%. Alignment coverages average 71–94% within their respective genomic groups (e.g., *C. sputigena*, *C. gingivalis*). This analysis supports genome-wide synteny and low contamination in TD-prevalent MAGs (see Table S8).

**Figure S8:** **Radial gene-detection heatmaps for *Capnocytophaga* MAGs across oral sites**. Radial gene-detection heatmaps for five tongue dorsum-prevalent *Capnocytophaga* metagenome-assembled genomes (MAGs) illustrating gene presence across oral sites. Each plot represents one MAG, showing detection of genes in the top 30 metagenomic samples per site ranked by breadth of coverage: supragingival plaque (SUPP; cyan), tongue dorsum (TD; purple), and buccal mucosa (BM; green). Genes are detected if ≥90% of nucleotides have ≥1X coverage (colored by site) or undetected (black), with a 90% threshold ensuring reliable detection. Genes are ordered by detection frequency, and samples within each site are ordered inward to outward by proportion of genes detected. Outer rings indicate gene categories: genus core (blue), genus accessory (brown), genus singleton (gold); species core (blue), species accessory (brown), species singleton (gold). Plots confirm 44–56% detection of genus- and species-specific core genes in TD samples.

**Figure S9**: Detection threshold sensitivity across *Capnocytophaga* genomic groups. Line plots illustrating the prevalence (proportion of samples with detection) of *Capnocytophaga* genomic groups across oral sites at varying thresholds for detection as measured by breadth of the genome (25%, 40%, 50%, 60%) covered by recruited reads. Each panel corresponds to a distinct genomic group, labeled by species or Human Microbial Taxon (HMT) designation, with the number of reference genomes in the group indicated in parentheses (n). The x-axis displays oral sites in abbreviated form: BM = buccal mucosa; HP = hard palate; KG = keratinized gingiva; Perio = periodontal pocket; PT = palatine tonsils; SUBP = subgingival plaque; SUPP = supragingival plaque; SV = saliva; TD = tongue dorsum; TH = throat. Prevalence on the y-axis represents the proportion of samples per site in which at least one genome within the group exceeded the specified breadth-of-coverage threshold, based on metagenomic read mapping to 117 dereplicated reference genomes across 1,321 oral samples (1,297 from the Human Microbiome Project and 24 from periodontal pockets). Lines and points are colored by threshold as indicated in the legend, demonstrating the robustness of habitat distribution patterns to threshold variation.
