# Supplementary material for: Spatial ecology of the Capnocytophaga genus in the human oral cavity
Source: Microbiol Spectr. 2026 Apr 30;14(6):e03626-25. doi: 10.1128/spectrum.03626-25 (PMC13228040; doi:10.1128/spectrum.03626-25)
Supplement: Supplemental figures — Fig. S1 to S9. [file spectrum.03626-25-s0002.pdf]

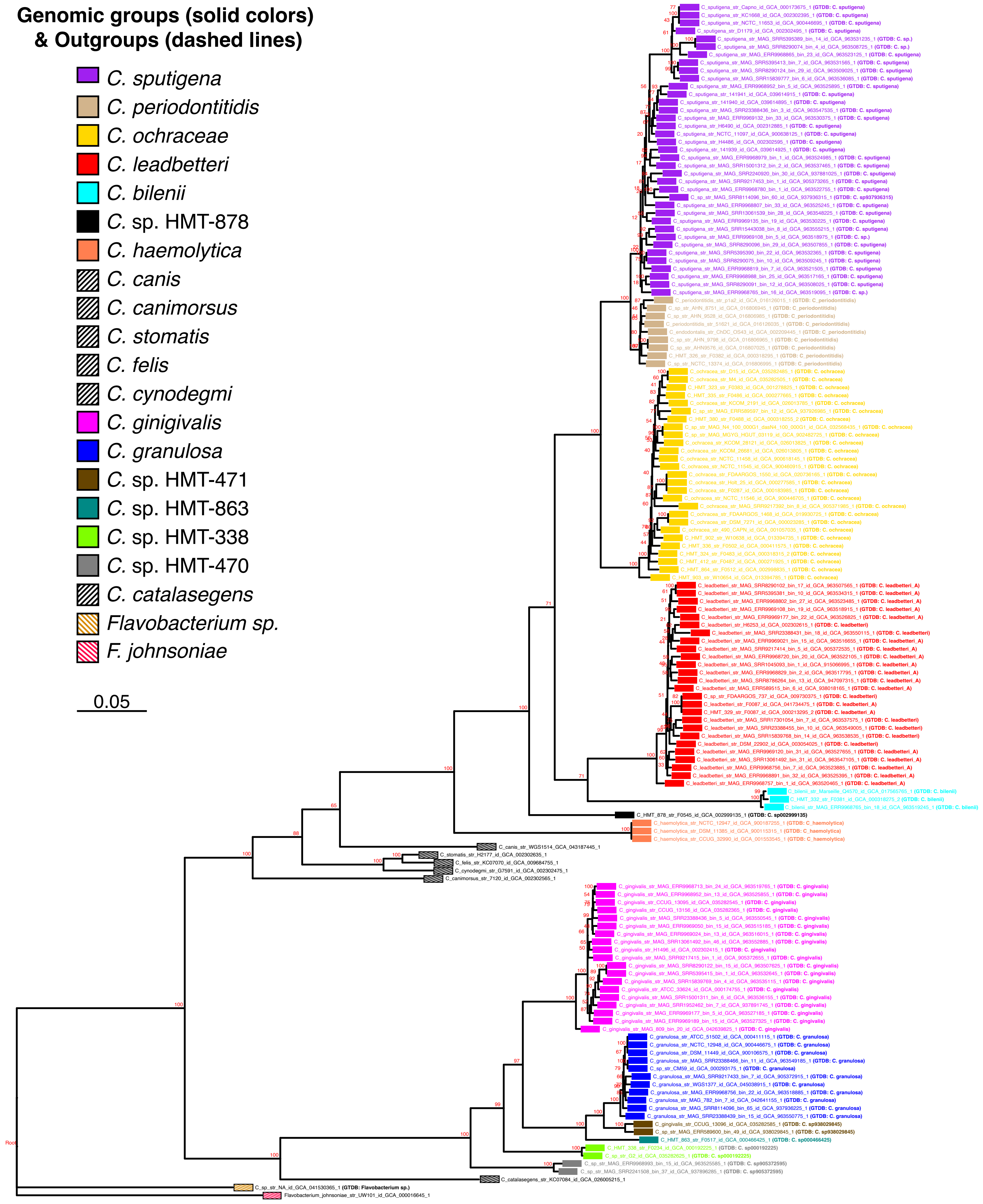

Genomic groups (solid colors)
& Outgroups (dashed lines)
C. sputigena
C. periodontitidis
C. ochraceae
C. leadbetteri
C. bilenii
C. sp. HMT-878
C. haemolytica
C. canis
C. canimorsus
C. stomatis
C. felis
C. cynodegmi
C. ginigivalis
C. granulosa
C. sp. HMT-471
C. sp. HMT-863
C. sp. HMT-338
C. sp. HMT-470
C. catalasegens
Flavobacterium sp.
F. johnsoniae
0.05

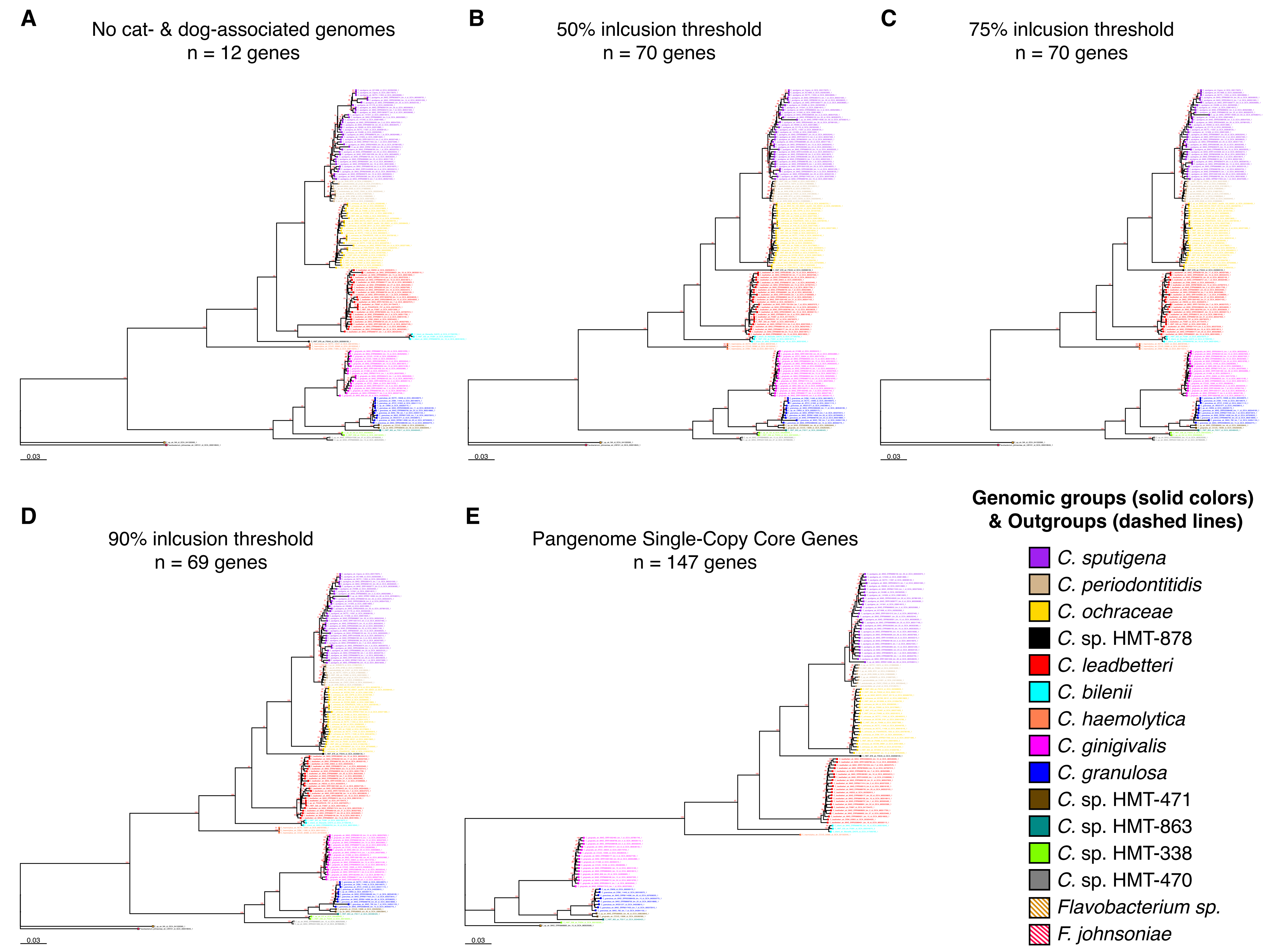
A
No cat- & dog-associated genomes
n = 12 genes
0.03
B
50% inlcusion threshold
n = 70 genes
0.03
C
75% inlcusion threshold
n = 70 genes
0.03
D
90% inlcusion threshold
n = 69 genes
0.03
E
Pangenome Single-Copy Core Genes
n = 147 genes
0.03
Genomic groups (solid colors)
& Outgroups (dashed lines)
C. sputigena
C. periodontitidis
C. ochraceae
C. sp. HMT-878
C. leadbetteri
C. bilenii
C. haemolytica
C. ginigivalis
C. granulosa
C. sp. HMT-471
C. sp. HMT-863
C. sp. HMT-338
C. sp. HMT-470
Flavobacterium sp.
F. johnsoniae

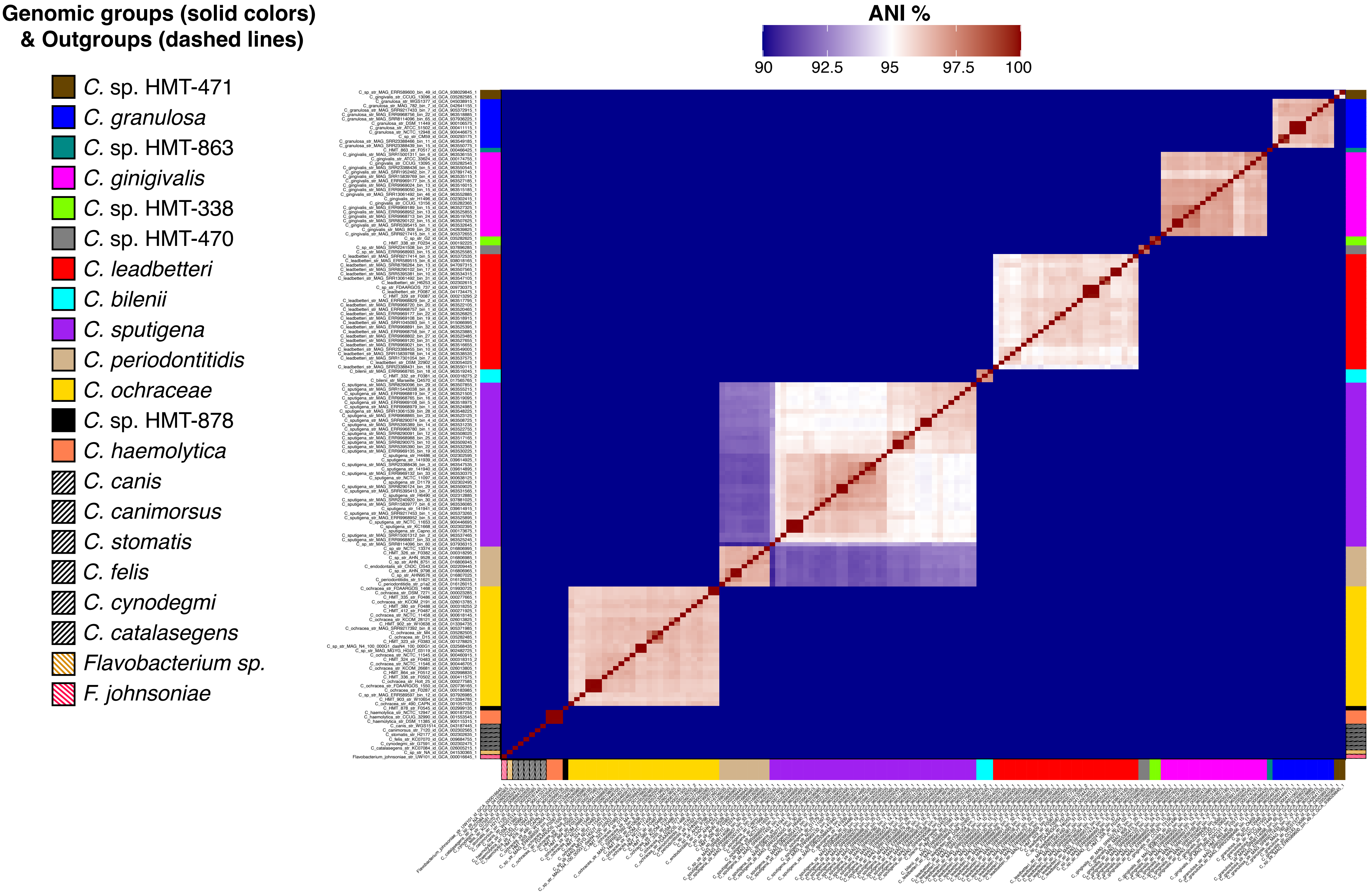

Genomic groups (solid colors) & Outgroups (dashed lines)
C. sp. HMT-471
C. granulosa
C. sp. HMT-863
C. ginigivalis
C. sp. HMT-338
C. sp. HMT-470
C. leadbetteri
C. bilenii
C. sputigena
C. periodontitidis
C. ochraceae
C. sp. HMT-878
C. haemolytica
C. canis
C. canimorsus
C. stomatis
C. felis
C. cynodegmi
C. catalasegens
Flavobacterium sp.
F. johnsoniae
ANI %
90
92.5
95
97.5
100

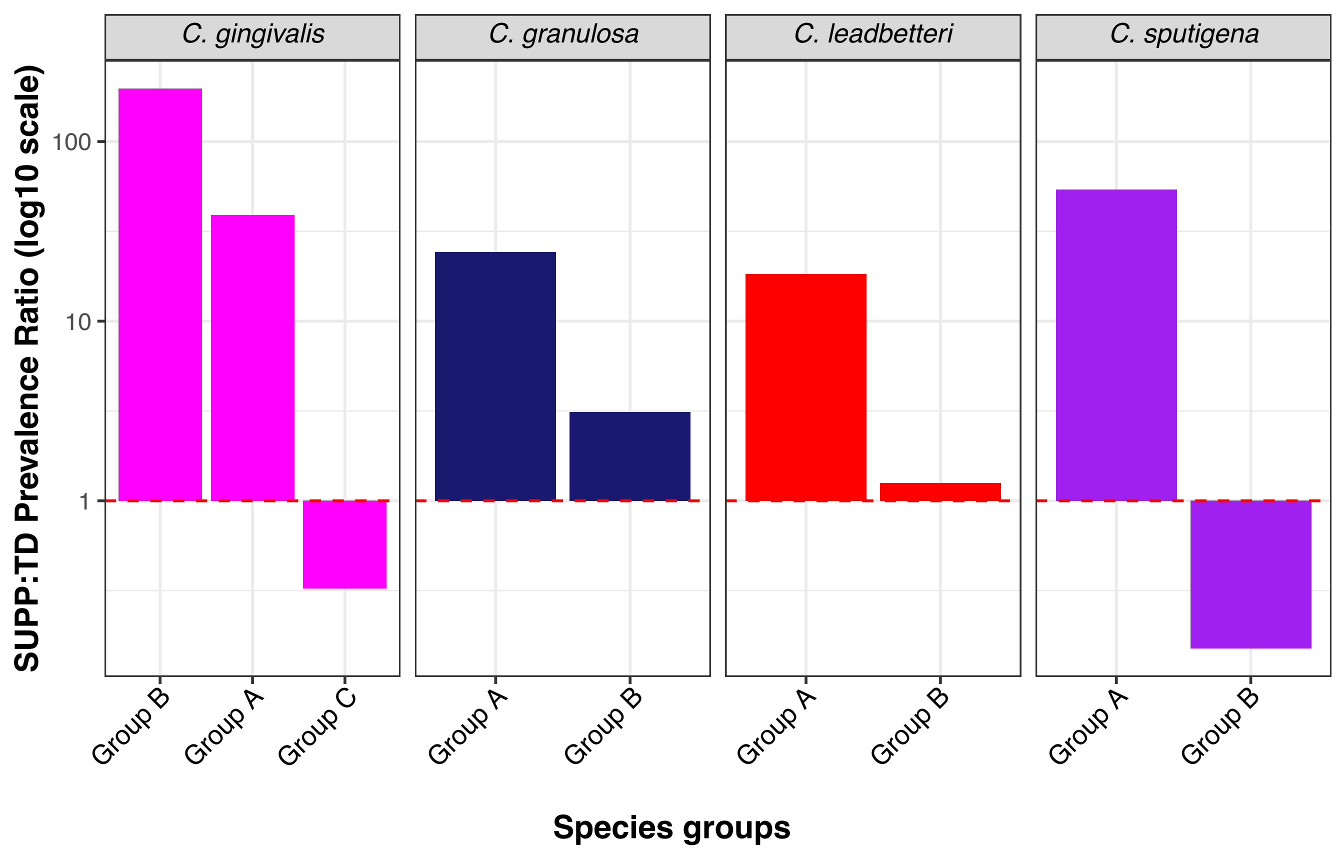

C. gingivalis
C. granulosa
C. leadbetteri
C. sputigena
SUPP:TD Prevalence Ratio (log10 scale)
100
10
1
Group B
Group A
Group C
Group A
Group B
Group A
Group B
Group A
Group B
Species groups

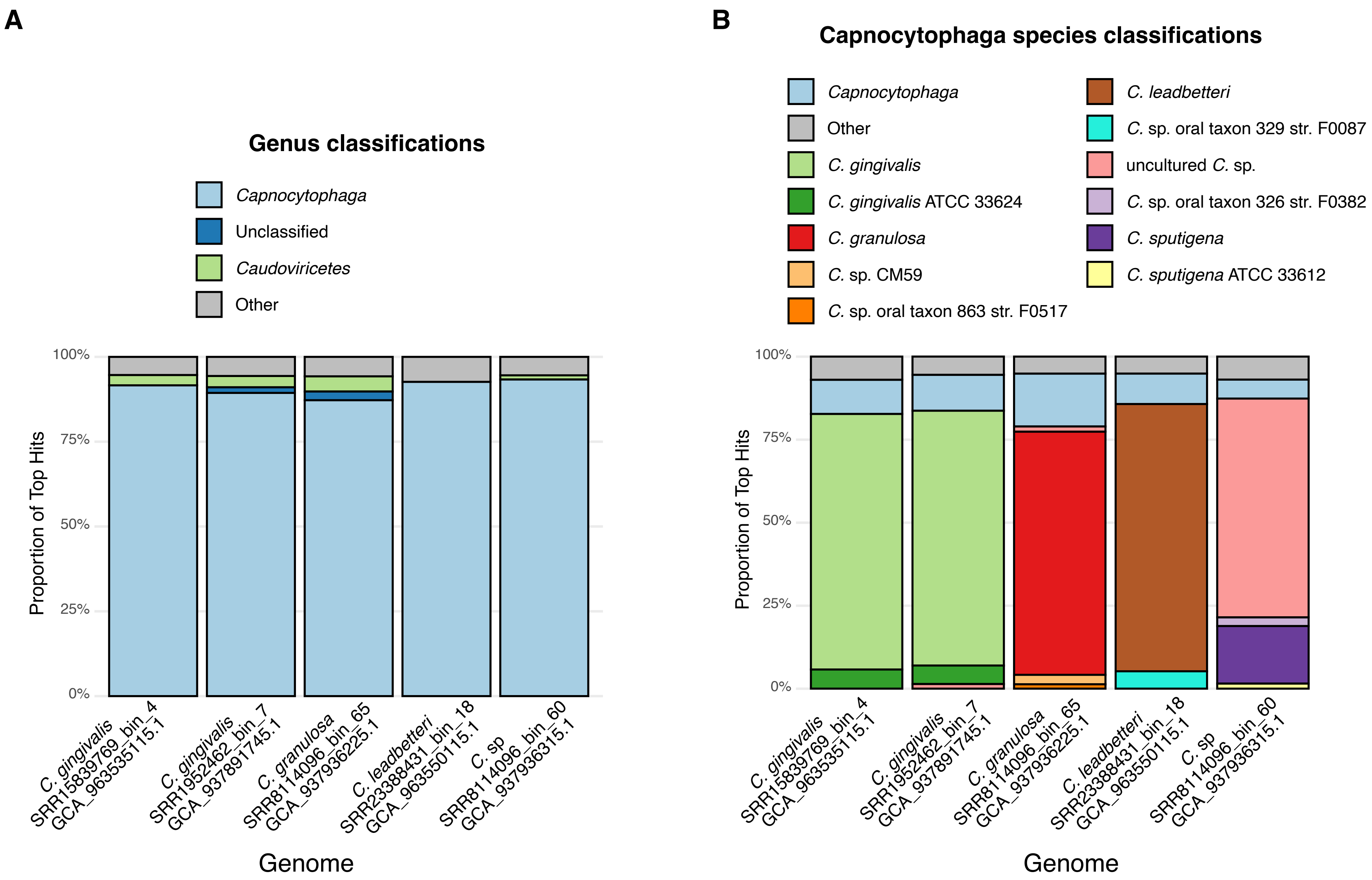
A
Genus classifications
Capnocytophaga
Unclassified
Caudoviricetes
Other
100%
75%
50%
25%
0%
Proportion of Top Hits
C. gingivalis SRR15839769_bin_4 GCA_963535115.1
C. gingivalis SRR1952462_bin_7 GCA_937891745.1
C. granulosa SRR8114096_bin_65 GCA_937936225.1
C. leadbetteri SRR23388431_bin_18 GCA_963550115.1
C. sp SRR8114096_bin_60 GCA_937936315.1
Genome
B
Capnocytophaga species classifications
Capnocytophaga
Other
C. gingivalis
C. gingivalis ATCC 33624
C. granulosa
C. sp. CM59
C. sp. oral taxon 863 str. F0517
C. leadbetteri
C. sp. oral taxon 329 str. F0087
uncultured C. sp.
C. sp. oral taxon 326 str. F0382
C. sputigena
C. sputigena ATCC 33612
100%
75%
50%
25%
0%
Proportion of Top Hits
C. gingivalis SRR15839769_bin_4 GCA_963535115.1
C. gingivalis SRR1952462_bin_7 GCA_937891745.1
C. granulosa SRR8114096_bin_65 GCA_937936225.1
C. leadbetteri SRR23388431_bin_18 GCA_963550115.1
C. sp SRR8114096_bin_60 GCA_937936315.1
Genome

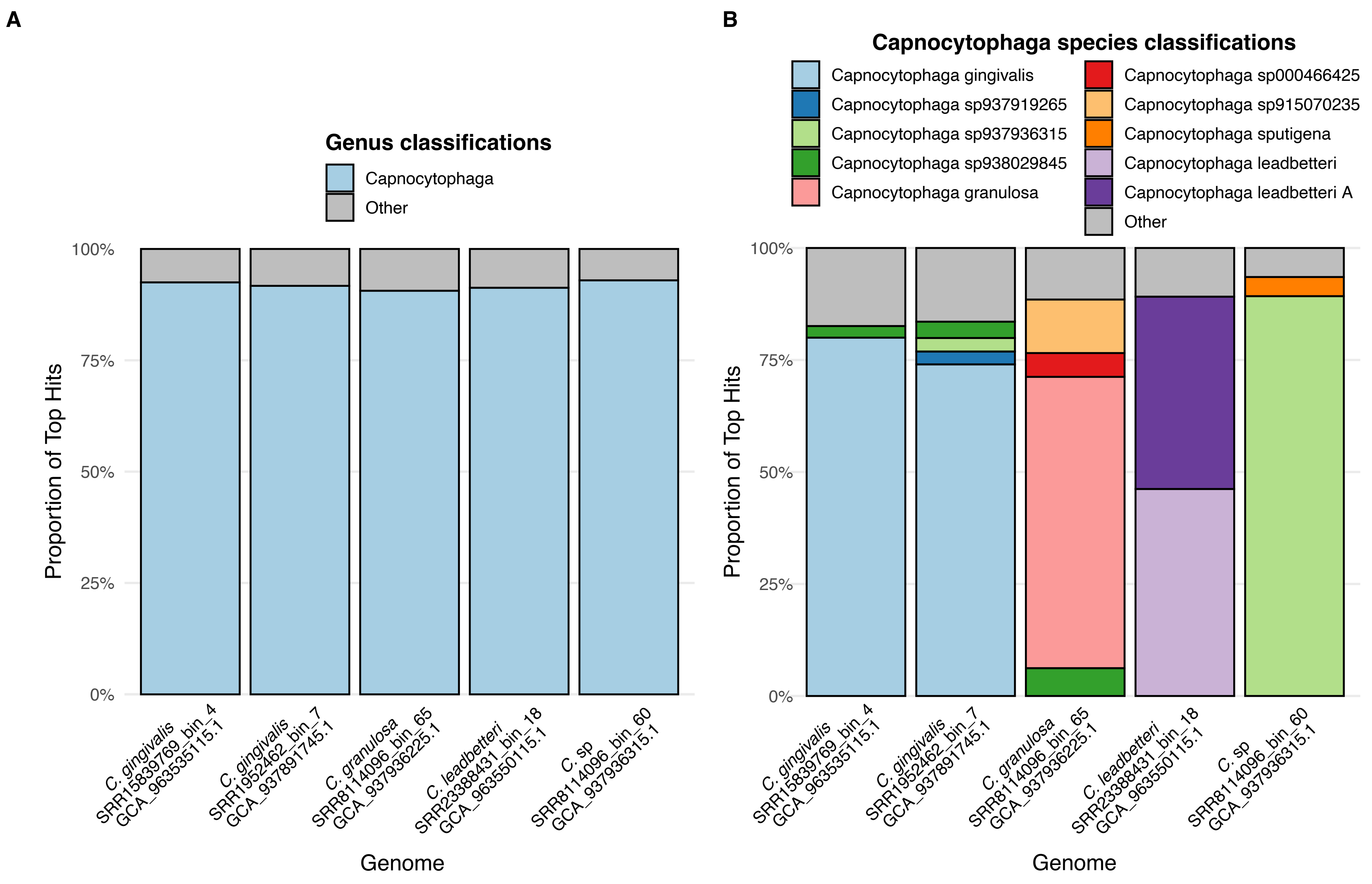
A
Genus classifications
Capnocytophaga
Other
100%
75%
50%
25%
0%
Proportion of Top Hits
C. gingivalis SRR15839769_bin_4 GCA_963535115.1
C. gingivalis SRR1952462_bin_7 GCA_937891745.1
C. granulosa SRR8114096_bin_65 GCA_937936225.1
C. leadbetteri SRR23388431_bin_18 GCA_963550115.1
C. sp SRR8114096_bin_60 GCA_937936315.1
Genome
B
Capnocytophaga species classifications
Capnocytophaga gingivalis
Capnocytophaga sp937919265
Capnocytophaga sp937936315
Capnocytophaga sp938029845
Capnocytophaga granulosa
Capnocytophaga sp000466425
Capnocytophaga sp915070235
Capnocytophaga sputigena
Capnocytophaga leadbetteri
Capnocytophaga leadbetteri A
Other
100%
75%
50%
25%
0%
Proportion of Top Hits
C. gingivalis SRR15839769_bin_4 GCA_963535115.1
C. gingivalis SRR1952462_bin_7 GCA_937891745.1
C. granulosa SRR8114096_bin_65 GCA_937936225.1
C. leadbetteri SRR23388431_bin_18 GCA_963550115.1
C. sp SRR8114096_bin_60 GCA_937936315.1
Genome

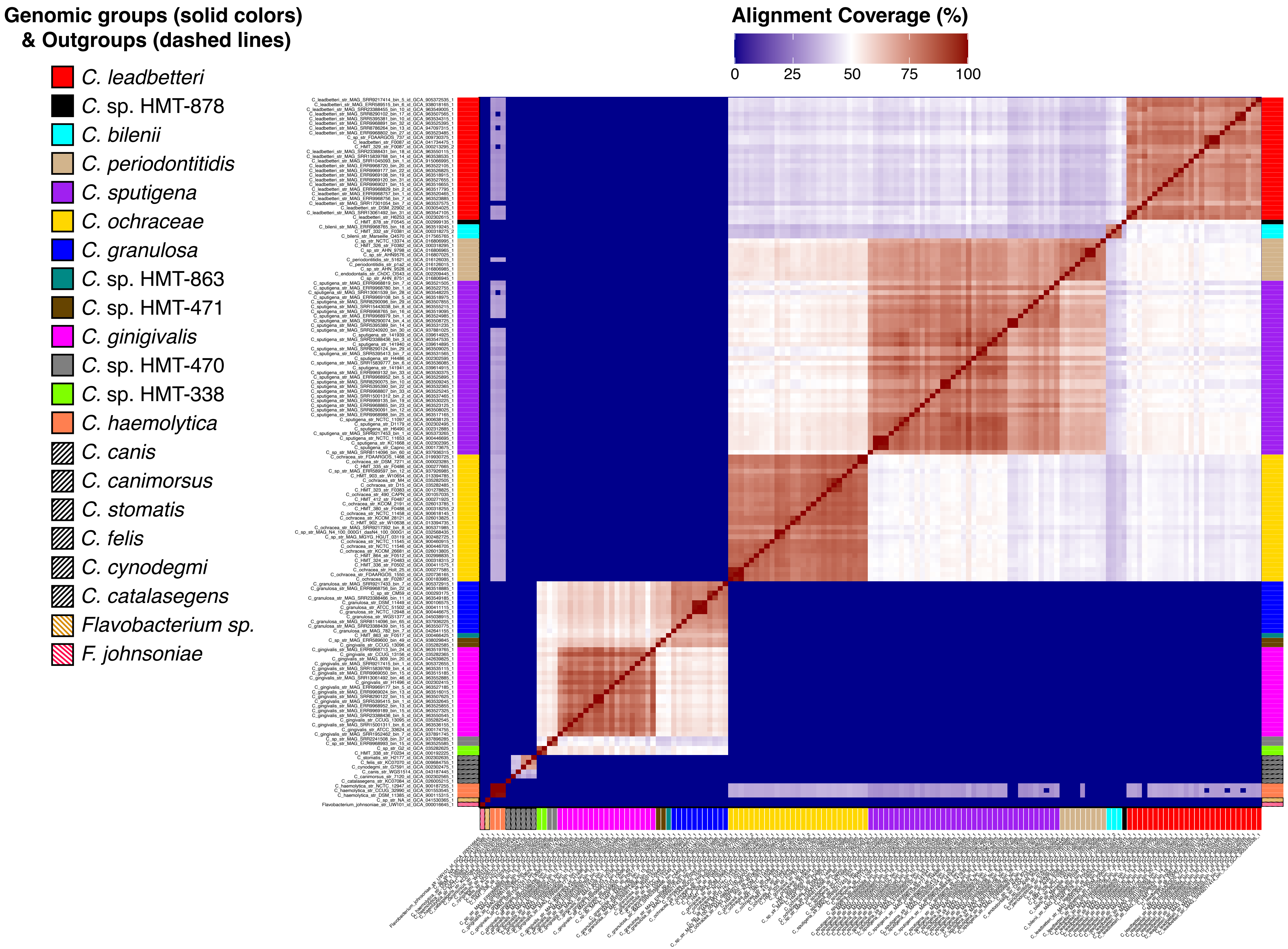
Genomic groups (solid colors)
& Outgroups (dashed lines)
C. leadbetteri
C. sp. HMT-878
C. bilenii
C. periodontitidis
C. sputigena
C. ochraceae
C. granulosa
C. sp. HMT-863
C. sp. HMT-471
C. ginigivalis
C. sp. HMT-470
C. sp. HMT-338
C. haemolytica
C. canis
C. canimorsus
C. stomatis
C. felis
C. cynodegmi
C. catalasegens
Flavobacterium sp.
F. johnsoniae
Alignment Coverage (%)
0
25
50
75
100

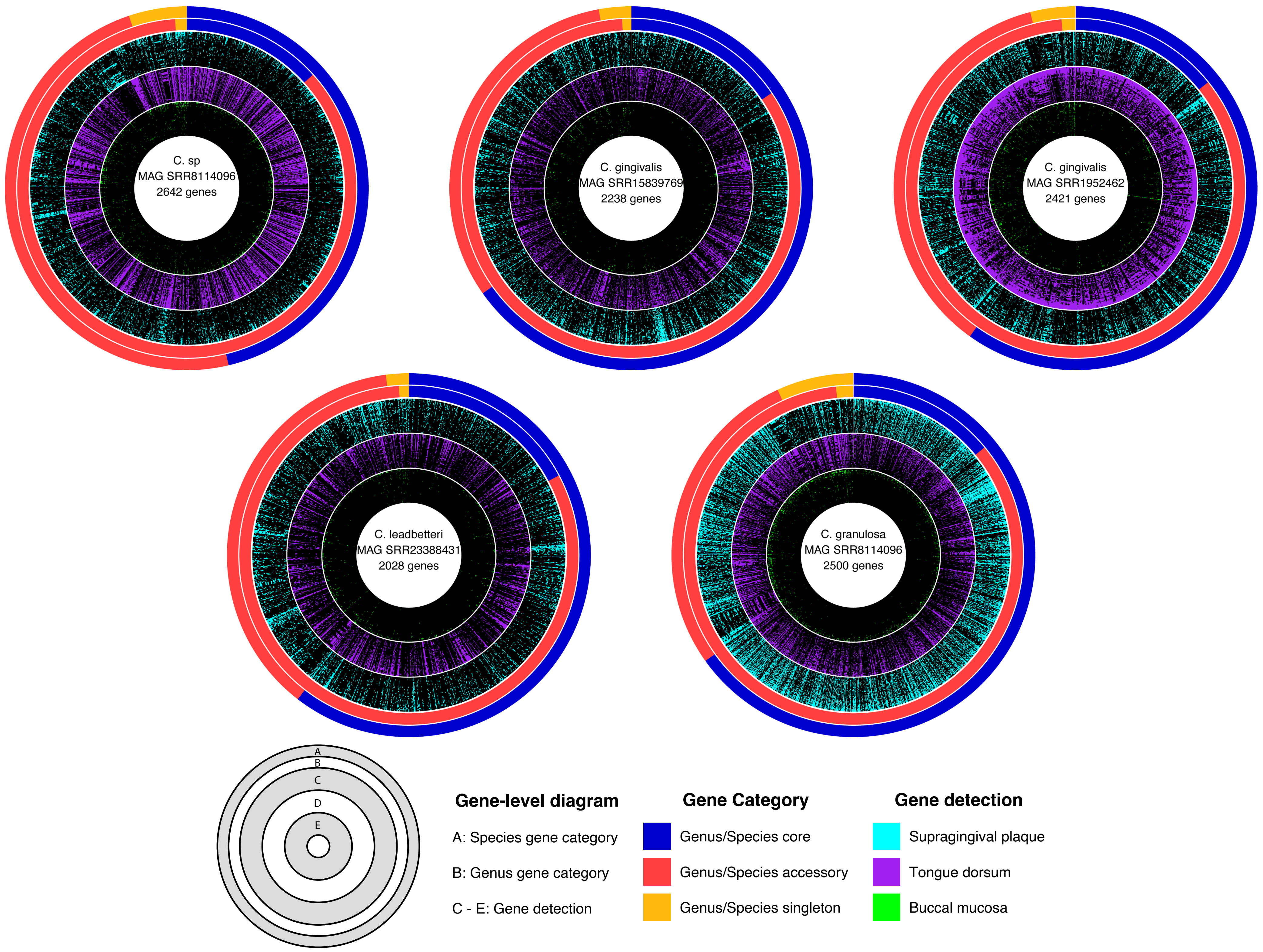
C. sp
MAG SRR8114096
2642 genes
C. gingivalis
MAG SRR15839769
2238 genes
C. gingivalis
MAG SRR1952462
2421 genes
C. leadbetteri
MAG SRR23388431
2028 genes
C. granulosa
MAG SRR8114096
2500 genes
A
B
C
D
E
Gene-level diagram
A: Species gene category
B: Genus gene category
C - E: Gene detection
Gene Category
Genus/Species core
Genus/Species accessory
Genus/Species singleton
Gene detection
Supragingival plaque
Tongue dorsum
Buccal mucosa

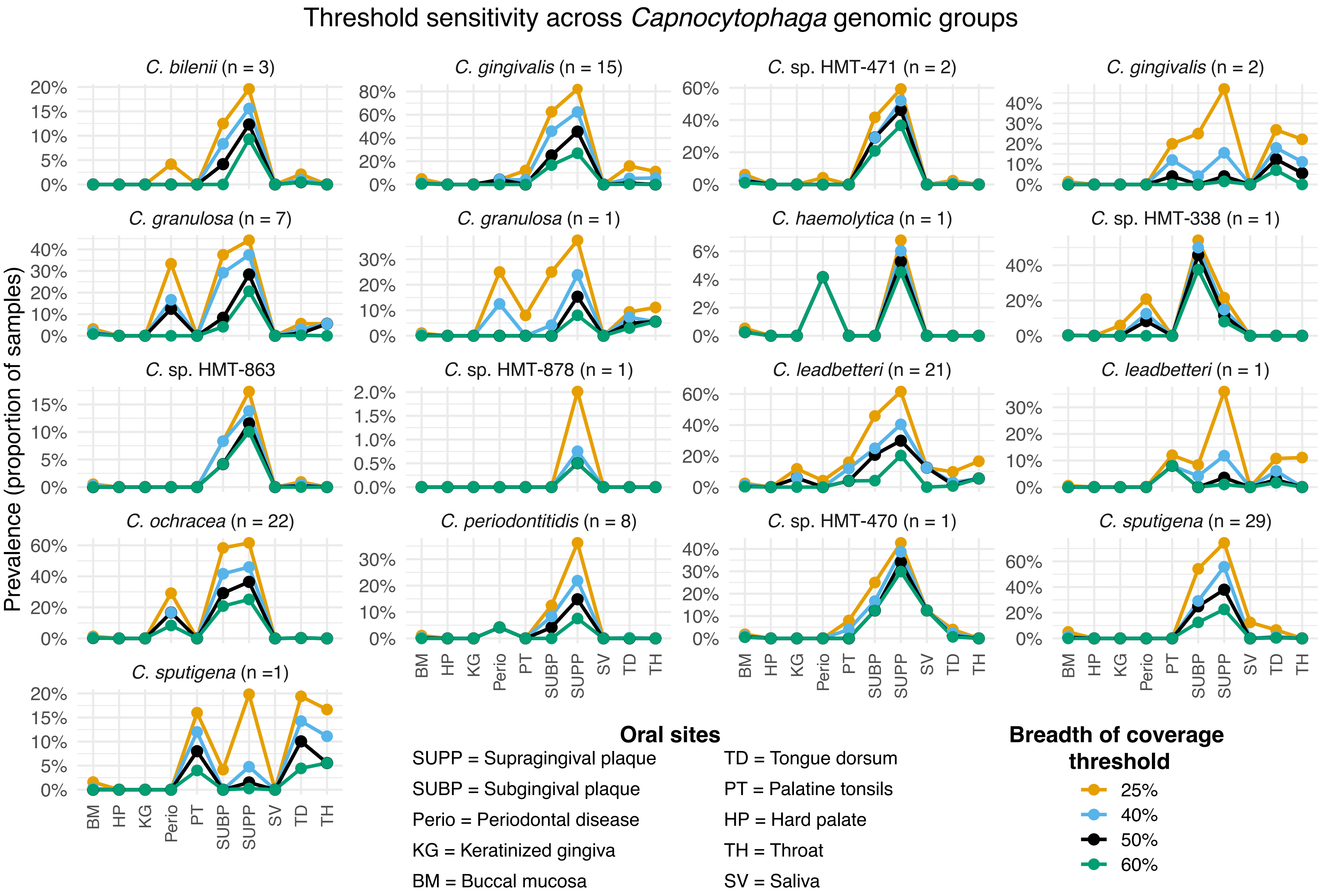
Threshold sensitivity across Capnocytophaga genomic groups
C. bilenii (n = 3)
C. gingivalis (n = 15)
C. sp. HMT-471 (n = 2)
C. gingivalis (n = 2)
C. granulosa (n = 7)
C. granulosa (n = 1)
C. haemolytica (n = 1)
C. sp. HMT-338 (n = 1)
C. sp. HMT-863
C. sp. HMT-878 (n = 1)
C. leadbetteri (n = 21)
C. leadbetteri (n = 1)
C. ochracea (n = 22)
C. periodontitidis (n = 8)
C. sp. HMT-470 (n = 1)
C. sputigena (n = 29)
C. sputigena (n =1)
Prevalence (proportion of samples)
BM
HP
KG
Perio
PT
SUBP
SUPP
SV
TD
TH
Oral sites
SUPP = Supragingival plaque
SUBP = Subgingival plaque
Perio = Periodontal disease
KG = Keratinized gingiva
BM = Buccal mucosa
TD = Tongue dorsum
PT = Palatine tonsils
HP = Hard palate
TH = Throat
SV = Saliva
Breadth of coverage threshold
25%
40%
50%
60%
