# Supplementary material for: Spatial ecology of the Capnocytophaga genus in the human oral cavity
Source: Microbiol Spectr. 2026 Apr 30;14(6):e03626-25. doi: 10.1128/spectrum.03626-25 (PMC13228040; doi:10.1128/spectrum.03626-25)
Supplement: Supplemental material — Supplemental text S1 to S3. [file spectrum.03626-25-s0003.docx]

**Supplemental Text**

**Supplemental Text S1: Phylogenetic Analysis**

A phylogenetic analysis of 144 *Capnocytophaga* genomes was conducted using the bacterial_71 conserved gene collection (4), with modifications to assess the impact of gene selection and outgroup inclusion on tree topology. Initially, a strict criterion requiring all 71 genes to be present in every genome (100% gene inclusion threshold) resulted in only 12 genes for analysis (Figure S1). We thus constructed phylogenetic trees for 5 different modifications – 1) Exclusion of cat-and dog-associated genomes, 2 - 4) exclusion of cat-and dog-associated genomes along with relaxing the gene inclusion threshold to presence in 50%, 75%, or 90% of genomes, 5) phylogeny based on pangenome single-copy core genes. For each modification, amino acid sequences were extracted from the genomes database with the Anvi’o program anvi-get-sequences-for-hmm-hits. Sequences were aligned using MUSCLE (v3.8.1551) (5)and concatenated into a FASTA file for tree construction. Alignments were trimmed using trimAl (6) with the setting ‘-gt 0.5’ to remove positions with gaps in more than 50% of sequences. Maximum likelihood phylogenetic trees were computed using IQ-TREE (7) with the WAG substitution model (8) and 1000 bootstrap replicates for support. A type strain genome for *Flavobacterium* *johnsoniae* (strain UW101; GCA_000016645.1) was included to root the trees.

Removing outgroup genomes, such as a *C*. sp. (GCA_041530365.1), which is likely a *Flavobacterium* as classified by GTDB, a *Flavobacterium* *johnsoniae* strain UW101 (GCA_000016645.1), and cat- and dog-associated genomes, did not significantly alter the gene count, indicating that gene loss was driven by variability among human-associated genomes. Relaxing the gene inclusion threshold to presence in 50%, 75%, or 90% of genomes increased the gene set to 70, 70, and 69 genes, respectively, with minimal gene exclusions (e.g., Exonuc_VII_L and Ribosomal_S20p at 90%). Across all scenarios, phylogenetic trees exhibited only minor within-clade adjustments, with no biologically meaningful changes to overall genomic groups, confirming the robustness of the phylogenetic structure regardless of gene set size or outgroup inclusion (Figure S2). Additionally, a phylogeny constructed from 147 single-copy core genes (SCGs) extracted from the human-associated genome pangenome (n = 117 genomes) recovered the same genomic groups as the bacterial_71-based phylogenies, with one exception: *C*. sp. MAG SRR8114096 bin 60 (GCA_937936315.1) shifted from the *C. sputigena* clade to a distinct position, but most closely related to *C. sputigena*.

**Supplemental Text S2: Detailed Composition of Genomic Groups**

One genomic group contained all *C. sputigena*, with one metagenome-assembled genome (MAG; GCA_937936315.1) appearing marginally distinct, embedded within the *C. sputigena* clade in the phylogeny, but sharing 92.8% to 93.7% ANI with other *C. sputigena* genomes, and classified by GTDB as C. sp937936315. A second genomic group contains all *C. granulosa* genomes, plus one genome classified in NCBI only to genus. Another genomic group contains all *C. ochracea* genomes, including genomes classified only to genus, and genomes with previous HMT designations in NCBI that are now classified as *C. ochracea* in HOMD. Genomes for *C. periodontitidis* grouped with genomes labeled in NCBI as *C*. *endodontalis*, and HMT-326 and genomes classified only to genus. All *C. leadbetteri* genomes formed a genomic group with the addition of one genome classified only to genus. A genomic group contains all three *C. bilenii* genomes. Single-genome (Singleton) genomic groups were identified for *C. haemolytica*, HMT-470, HMT-878, and HMT-863, and two-genome groups for HMT-338 and HMT-471, with one genome in the latter likely misclassified as *C. gingivalis* in NCBI. Collectively, these genomic groups clarify genome relatedness, enabling finer-scale analysis of habitat distributions across the oral cavity.

**Supplemental Text S3: Analysis of Contamination for TD-prevalent MAGs**

Strain-level genome coverage analysis reveals potential habitat preferences for a subset of metagenome-assembled genomes (MAGs) with elevated coverage in tongue dorsum compared to dental plaque sites, suggesting intra-species diversity (Fig. 2). However, the high community complexity from which MAGs are constructed increases the likelihood of contamination in the genome assembly, which could result in misleading mapping results. To determine whether these MAGs represent *Capnocytophaga* lineages genuinely adapted to the tongue dorsum or chimeric artifacts, we performed detailed contamination checks of the assembled genomes.

Contig-level taxonomic consistency was assessed for five focal *Capnocytophaga* MAGs using MMSeqs2 Version 17-b804f (1) and GTDB-tk Version 2.3.0 (2). For the MMSeqs2 analysis, we downloaded pre-built reference databases (GTDB for bacterial genomes and NCBI non-redundant (3) for a broader protein database) and classified contigs from each MAG’s FASTA file using mmseqs easy-taxonomy with default settings. This process compared each contig sequence against the databases, identifying the best match based on alignment quality. The easy-taxonomy program assigned taxonomic labels to each contig sequence for each MAG based on the lowest common ancestor of all equal-scoring top hits, the results of which are summarized in Supplemental data: Table S8. We then examined the top hit results, which were used to assign taxonomic labels, by plotting the distributions of taxonomic classifications for each top hit. We filtered top hits with at least 75% sequence identity to increase confidence in the matches, grouped them by genus (e.g., simplifying "*Capnocytophaga* *leadbetteri*" to "*Capnocytophaga*"), and calculated the proportion of top hits assigned to each genus per MAG. For top hits classified as *Capnocytophaga*, we further broke down the species-level assignments. These proportions were plotted as stacked bar charts, showing the distribution of classifications across each MAG, with anything below 1-2.5% grouped as "Other" for clarity (Fig. S5 and Fig. S6). These methods helped us spot any significant presence of non-*Capnocytophaga* taxa, which could indicate contamination.

Complementary taxonomic classification of contigs via GTDB-tk validated consistency for the five *Capnocytophaga* MAGs. We extracted contig sequences from the previously constructed Anvi’o contigs database that we built for the dereplicated set of genomes (N = 117). We then split the contig nucleotide sequences into separate FASTA files for each MAG. GTDB-tk classify_wf was then run on these files, classifying each contig against the R220 bacterial reference database. To classify relatively short contig sequences using GTDB-tk classify_wf, we set the parameter --min_perc_aa to zero. This parameter controls filtering of query sequences below a specified percentage of amino acids in the concatenated set of aligned marker genes. Setting it to zero effectively negates this filter step, enabling classification for sequences containing a low number of marker genes. We merged these GTDB-tk classifications with the MMSeqs2 results (both GTDB and NR) into a single table for each MAG, adding contig length and GC content for additional context. This combined file let us compare classifications across tools per contig, revealing any inconsistencies that might suggest a mix of species within a MAG.

Gene-level coverage analysis evaluated contamination in the five tongue dorsum-prevalent *Capnocytophaga* MAGs. The anvi-summarize program with the --init-gene-coverages flag produced gene-level breadth of coverage data frames for each genome in the reference set. To facilitate visualization of the results, we focused on a subset of samples from tongue dorsum (TD), supragingival plaque (SUPP), and buccal mucosa (BM), selecting for each the top 30 samples with the highest genome-wide breadth of coverage for each of the five MAGs. These data were then linked to a *Capnocytophaga* genus-level pangenome, in which we categorized genes as core, accessory, or singleton based on their prevalence in genomes across the genus and species. For each MAG, we calculated the proportion of genes detected at 90% breadth thresholds (i.e., genes covered across at least 90% of their length). We visualized these in radial plots, where rings represented samples and colors indicated detection status, with separate bands showing genus- and species-level gene categories (Fig. S7).

To further evaluate the possibility of chimeric contamination in the five tongue dorsum-prevalent metagenome-assembled genomes (MAGs), we performed chimerism detection using the Genome UNClutterer (GUNC; Orakov et al., 2021). GUNC assesses taxonomic homogeneity across predicted genes by aligning them against a comprehensive prokaryotic reference database (ProGenomes 2.1 in this analysis) using DIAMOND, assigning lowest common ancestor (LCA) taxonomic labels to each gene, and quantifying discordance via a weighted principal component analysis-derived clade separation score (CSS). The analysis was executed with default parameters on the five focal MAGs (GCA_937936315.1, GCA_937891745.1, GCA_963535115.1, GCA_963550115_1, and GCA_937936225_1).

GUNC reports several key metrics: (i) the clade separation score (CSS), where values >0.45 at any taxonomic rank are considered indicative of significant chimerism; (ii) the effective number of additional phylogenetic clades beyond the dominant lineage required to explain gene taxonomic heterogeneity; (iii) the proportion of predicted genes confidently assigned to the major clade(s); (iv) the estimated proportion of the genome attributable to non-dominant lineages (contamination portion); and (v) reference representation and mean hit identity scores, which reflect database coverage and alignment quality of the dominant lineage. In clean, non-chimeric genomes, CSS is typically near 0.0 at higher ranks (genus and above), the proportion of genes retained in major clades exceeds 0.90–0.95, and the effective number of surplus clades is low (<0.5–1.0). Elevated CSS with low gene retention or poor reference representation may instead reflect database incompleteness, extensive horizontal gene transfer, or strain-level heterogeneity rather than true multi-lineage chimerism.

Results of the GUNC analysis are provided in Table S10. All five MAGs exhibited CSS = 0.0 at genus and higher taxonomic ranks, with species-rank CSS values ranging from 0.04 to 0.17 (well below the 0.45 threshold). The proportion of genes assigned to major clades remained high (0.91–0.99 across ranks; 0.91–0.92 at species level), mean hit identity was strong (0.92–0.96), and reference representation scores ranged from 0.84 to 0.91. All genomes passed GUNC filtering at every taxonomic rank. These patterns indicate taxonomic coherence, negligible chimerism, and high-quality assembly consistent with authentic *Capnocytophaga* strain-level variants rather than chimeric artifacts.

**References**

1. Steinegger M, Söding J. 2017. MMseqs2 enables sensitive protein sequence searching for the analysis of massive data sets. Nat Biotechnol 35:1026–1028.

2. Chaumeil P-A, Mussig AJ, Hugenholtz P, Parks DH. 2020. GTDB-Tk: a toolkit to classify genomes with the Genome Taxonomy Database. Oxford University Press.

3. Sayers EW, Bolton EE, Brister JR, Canese K, Chan J, Comeau DC, Connor R, Funk K, Kelly C, Kim S, Madej T, Marchler-Bauer A, Lanczycki C, Lathrop S, Lu Z, Thibaud-Nissen F, Murphy T, Phan L, Skripchenko Y, Tse T, Wang J, Williams R, Trawick BW, Pruitt KD, Sherry ST. 2022. Database resources of the national center for biotechnology information. Nucleic Acids Res 50:D20–D26.

4. Lee MD. 2019. GToTree: a user-friendly workflow for phylogenomics. Bioinformatics 35:4162–4164.

5. Edgar RC. 2004. MUSCLE: multiple sequence alignment with high accuracy and high throughput. Nucleic Acids Res 32:1792–1797.

6. Capella-Gutiérrez S, Silla-Martínez JM, Gabaldón T. 2009. trimAl: a tool for automated alignment trimming in large-scale phylogenetic analyses. Bioinformatics 25:1972–1973.

7. Nguyen L-T, Schmidt HA, Von Haeseler A, Minh BQ. 2015. IQ-TREE: a fast and effective stochastic algorithm for estimating maximum-likelihood phylogenies. Mol Biol Evol 32:268–274.

8. Whelan S, Goldman N. 2001. A general empirical model of protein evolution derived from multiple protein families using a maximum-likelihood approach. Mol Biol Evol 18:691–699.
